# Druggability Studies of Benzene Sulfonamide Substituted Diarylamide (E3) as a Novel Diuretic

**DOI:** 10.3390/biomedicines13040992

**Published:** 2025-04-18

**Authors:** Hang Zhang, Shuyuan Wang, Nannan Li, Yue Xu, Zhizhen Huang, Yukun Zhang, Jing Li, Yinglin Zuo, Min Li, Runtao Li, Baoxue Yang

**Affiliations:** 1 Department of Pharmacology, School of Basic Medical Sciences, Peking University, Beijing 100191, China; hangzhang@bjmu.edu.cn (H.Z.); shuyuan0127@outlook.com (S.W.); 1610305107@pku.edu.cn (N.L.); huangzz@pku.edu.cn (Z.H.); leemin@bjmu.edu.cn (M.L.); 2 Division of Pharmaceutics and Pharmacology, College of Pharmacy, The Ohio State University, Columbus, OH 43210, USA; xu.5020@osu.edu; 3 Chongqing Key Laboratory of Development and Utilization of Genuine Medicinal Materials in Three Gorges Reservoir Area, Chongqing 404120, China; yukun_zhang@cqtgmc.edu.cn; 4 The State Key Laboratory of Anti-Infective Drug Development, Sunshine Lake Pharma Co., Ltd., Dongguan 523871, China; lijing@hec.cn (J.L.); zuoyinglin@hec.cn (Y.Z.); 5 School of Pharmaceutical Sciences, Peking University, Beijing 100191, China; lirt@bjmu.edu.cn

**Keywords:** urea transporter inhibitor, diuretic, structure optimization, pharmacokinetic, safety evaluation, hyponatremia

## Abstract

**Background/Objectives**: Urea transporters (UTs) play an important role in the urine-concentrating mechanism and have been regarded as a novel drug target for developing salt-sparing diuretics. Our previous studies found that diarylamides 1H and 25a are specific UT inhibitors and have oral diuretic activity. However, these compounds necessitate further optimization and comprehensive druggability studies. **Methods**: The optimal compound was identified through structural optimization. Experiments were conducted to investigate its UT inhibitory activity and evaluate its diuretic effect. Furthermore, disease models were utilized to assess the compound’s efficacy in treating hyponatremia. Pharmacokinetic studies were performed to examine its metabolic stability, and toxicity tests were conducted to evaluate its safety. **Results**: Based on the chemical structure of compound 25a, we synthesized a novel diarylamide compound, E3, by introducing a benzenesulfonamide group into its side chain. E3 exhibited dose-dependent inhibition of UT at the nanomolar level and demonstrated oral diuretic activity without causing electrolyte excretion disorders in both mice and rats. Experiments on UT-B^−/−^ and UT-A1^−/−^ mice indicated that E3 enhances the diuretic effect primarily by inhibiting UT-A1 more effectively than UT-B. Furthermore, E3 displayed good metabolic stability and favorable pharmacokinetic characteristics. E3 significantly ameliorated hyponatremia through diuresis in a rat model. Importantly, E3 did not induce acute oral toxicity, subacute oral toxicity, genotoxicity, or cardiotoxicity. **Conclusions**: Our study confirms that E3 exerts a diuretic effect by specifically inhibiting UTs and has good druggability, which offers potential for E3 to be developed into a new diuretic for the treatment of hyponatremia.

## 1. Introduction

Diuretics are drugs that increase urine output by affecting the reabsorption and secretion of renal tubules, making them commonly used for the treatment of hypertension, heart failure, edema, and ascites, etc. [1,2,3]. However, traditional diuretics, such as loop, thiazide, and potassium-sparing diuretics, when used long term, may induce electrolyte disorders in the body [4,5,6]. Hyponatremia is the most common electrolyte disorder diagnosed in the hospital setting, which is a significant independent risk factor for in-hospital mortality [7,8,9]. Vasopressin V2 receptor (V2R) antagonists are effective in alleviating hyponatremia, but their hepatotoxicity was demonstrated in clinical trials [10]. Therefore, the search for novel diuretic targets and safer diuretics that avoid electrolyte disturbances is crucial.

Urea is a major solute in the hyperosmolar renal medulla and plays an important role in urinary concentration management [11,12,13]. Urea transporters (UTs) are membrane channel proteins that are specifically permeable to urea. UTs play an important role in maintaining intrarenal urea recycling, establishing a urea concentration gradient in the renal medullary tissue [14,15]. Several UT knockout mouse models demonstrate that UT knockout blocks the intrarenal urea recycling and decreases the urine concentration ability, thereby producing a diuretic effect, which suggests UT inhibitors can be developed into diuretics for long-term clinical use without causing electrolyte disorders [16,17].

Since 2012, Verkman’s team has identified various small-molecule UT inhibitors, mainly including phenylsulfoxyoxazole, benzenesulfonanilide, phthalazinamine, aminobenzimidazole, 8-Hydroxyquinolines, aminothiazolones, benzo-[1,3,5]-triazines, and triazolothienopyrimidine active compounds [18,19,20] (Figure S1A–H). Our research group also identified thienoquinoline compounds PU-14 and PU-48 (Figure S1I,J) and a thienopyridine compound CB-20 (Figure S1K) [21]. However, all these compounds exert diuretic activity with low druggability.

**Figure 1:**
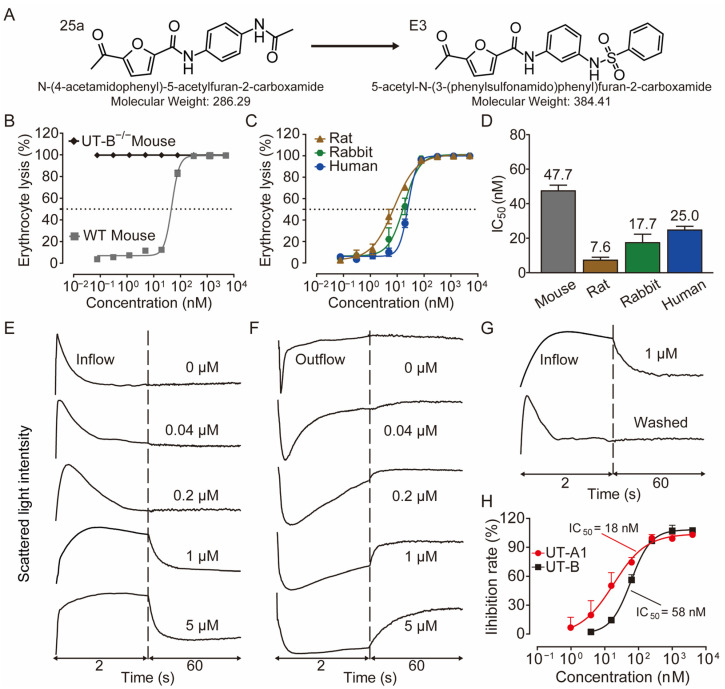
Inhibition activity of E3 on UT-B and UT-A1. (**A**) Chemical structure of 25a and E3. (**B**) Erythrocyte lysis percentage in wild-type (WT) and UT-B knockout (UT-B^−/−^) mice. (**C**) Erythrocyte lysis percentage in rats, rabbits, and humans. (**D**) IC_50_ value of E3 on UT-B-facilitated urea transport in mice, rats, rabbits, and humans. (**E**) Effect of E3 on UT-B-mediated urea influx, (**F**) effect of E3 on UT-B-mediated urea efflux, and (**G**) reversibility of UT-B inhibition were measured by stopped-flow light scattering. (**H**) Inhibition rate of E3 against UT-A1 and UT-B mediated urea permeability in UT-A1-MDCK and UT-B-MDCK cells. Data are presented as mean ± SEM (n = 3).

Recently, we found that diarylamides 1H (Figure S1L) and 25a (Figure 1A) showed superior diuretic effects in vivo without causing electrolyte imbalance in rats by oral administration [22,23,24]. It was found that 25a is effective in treating hyponatremia in the syndromes of the inappropriate antidiuretic hormone secretion (SIADH) model and cirrhosis ascites model [25,26]. However, the oral therapeutic dose of 25a in the disease models remains relatively high at 100 mg/kg, indicating a need for improvement in inhibitory activity and metabolic stability. Furthermore, it is crucial to note that the safety evaluation data for diarylamide compounds is lacking.

In this study, we derived a new diarylamide UT inhibitor, 5-acetyl-*N*-(3-(phenylsulfonamido)phenyl)furan-2-carboxamide (E3), based on the structural modification of 25a. E3 exhibited good inhibitory activities and selectivity for UT-A1 in vitro and in vivo. E3 demonstrated oral diuretic and therapeutic effects without significant toxicity. These experimental data suggest that E3 has the potential to be developed as a new diuretic for the treatment of hyponatremia.

## 2. Materials and Methods

### 2.1. Chemistry

All chemicals and solvents were obtained commercially and utilized without additional purification. NMR characterization was performed on a Bruker AVANCEIII spectrometer (Bruker, Karlsruhe, Germany) operating at 400 MHz (^1^H) and 100 MHz (^13^C). Chemical shifts are reported in δ (ppm) with peak multiplicities denoted as follows: s (singlet), d (doublet), t (triplet), q (quartet), m (multiplet), and br (broad). HRMS spectra were acquired by electrospray ionization (ESI) in positive ion mode or negative ion mode using Thermo Scientific Orbitrap Elite MS. The general procedure and schemes for the synthesis of intermediates and target compounds are shown in the Supplemental Materials.

### 2.2. Purity of Compound E3

Compound E3 employed in biological evaluations demonstrated 96.5% purity via HPLC (Agilent 1260 system; Agilent Technologies Inc., Palo Alto, CA, USA) using a ZORBAX Eclipse Plus C18 column (150 × 4.6 mm, 3.5 μm) with DAD detection at 254 nm. Mobile phases: (A) water and (B) acetonitrile under programmed gradient: 20% B (0 min) → 40% B (5 min) → 80% B (15 min) → 20% B (16 min).

### 2.3. Animals

C_57_BL/6 mice (20~22 g) and male Sprague–Dawley (SD) rats (200~220 g) were purchased from the Laboratory Animal Center of Peking University (Beijing, China). UT-B knockout (UT-B^−/−^) and UT-A1 knockout (UT-A1^−/−^) mice with a C_57_BL/6 genetic background were generated by targeted gene disruption as described previously [22]. Animals were housed under controlled conditions (25 ± 1 °C, 12h light/dark cycle) with ad libitum access to food and water. All experimental protocols (IACUC approval No. LA220354) strictly followed the ARRIVE guidelines (https://www.nc3rs.org.uk/arrive-guidelines (accessed on 3 November 2022)) and the National Research Council’s guide for the Care and Use of Laboratory Animals (https://olaw.nih.gov/policies-laws/guide-care-use-lab-animals (accessed on 3 November 2022)), in compliance with institutional ethics regulations at Peking University Health Science Center (approval date: 19 May 2020).

### 2.4. Blood Samples

Human venous blood was ethically obtained from healthy adult male volunteers following approval by the Ethics Committee of Peking University (Beijing, China). Rabbit blood was collected from male Japanese white rabbits by ear vein bleeding. Blood samples from male SD rats (orbital plexus puncture) and wild-type/UT-B^−/−^ mice (eyeball extirpation) were collected using 0.5% heparin anticoagulation for erythrocyte lysis and stopped-flow assays. Erythrocytes were isolated through sequential PBS washing (10 mM, pH 7.4) and centrifugation (2000× *g*, 10 min). All blood samples were processed within 12 h post-collection.

### 2.5. Erythrocyte Lysis Assay for Identifying UT-B Inhibition Activity

The erythrocyte lysis assay was modified from a method described previously [18]. Erythrocytes obtained from veins were diluted to a hematocrit value of 2% in hyperosmolar PBS containing 1.25 mol/L urea and 5 mmol/L glucose and incubated at r.t. for 2 h. Then, 99 μL erythrocyte suspension from a reservoir was added to each well of a 96-well round-bottom microplate, then 1 μL of the testing compound (500, 125, 31.25, 7.81, 1.95, 0.488, 0.122, 0.031, 0.0076 μmol/L dissolved in DMSO) was added to erythrocyte suspension and shook it up with microoscillator for 1 min. Following 5 min incubation, 20 μL erythrocyte suspension was transferred into 96-well black microplates preloaded with 180 μL isotonic PBS (10 mM, urea-free). Erythrocyte lysis was monitored at 710 nm optical density (OD710) for 6 min, with lysis rates calculated using control values from the same plate as Equation (1):

Lysis (%) = 100 × (A_neg_ − A_test_)/(A_neg_ − A_pos_) (1)

where A_test_ is the absorbance value from the test well, A_neg_ is from a negative no-lysis control well, and A_pos_ is from a positive full-lysis control.

### 2.6. Stopped-Flow Measurement of Erythrocyte Urea Permeability

Erythrocyte urea permeabilities were assessed via stopped-flow light scattering (SX20, Applied Photophysics, Leatherhead, UK) following established protocols [22]. Erythrocyte was acquired from rat blood and suspended in isotonic PBS (hematocrit 0.5%). Then, the erythrocyte was incubated with test compounds for 5 min and quickly mixed with 500 mmol/L urea dissolved in PBS. Following an initial osmotic shrinking phase, the kinetics of cell volume increase due to urea influx were measured by monitoring the time course of 90° scattered light intensity at 530 nm. The increase in cell volume resulted in a reduction in scattered light intensity. Keep samples and PBS at 4 °C to reduce the influence of free diffusion. To assay reversibility, compounds were added to erythrocytes for 5 min and then washed with PBS 3 times by 2000 r/min centrifugation before stopped-flow measurements. To determine inhibition on urea efflux, erythrocytes were incubated with 500 mmol/L urea in PBS for 2 h, then mixed with PBS without urea.

### 2.7. Transwell Assay of UT-A1/UT-B Inhibition

MDCK cells stably expressing rat UT-A1 or UT-B were cultured in DMEM supplemented with 10% FBS, and the mRNA levels of UT-A1 and UT-B were measured in our previous research [21]. Urea flux was assessed according to previously established methods. MDCK cells (2 × 10^5^ cells/cm^2^) were grown on 12 mm collagen-coated Costar Transwell inserts (0.4 μm pore size, Corning) for 4 d at 37 °C in the presence of 5% CO_2_. Once the cells on the apical side formed a tight monolayer (transepithelial resistance 1 kΩ/cm^2^), PBS (pH = 7.4, containing 10 μmol/L forskolin) with E3 or DMSO was added to the top (0.25 mL) and bottom (1 mL) compartments, and the cultures were incubated in the absence of urea for 30 min at 37 °C. As UT-B is located in the plasma membrane while UT-A1 resides in the cytoplasm, forskolin was used to stimulate the translocation of UT-A1 from the cytoplasm to the membrane for urea transport. Subsequently, the solution in the bottom compartment was replaced with PBS (pH 7.4, containing 10 μmol/L forskolin and E3 or DMSO) supplemented with 15 mmol/L urea. Apical fluid samples (5 μL) were collected at 0, 1, 3, 5, 10, 15, 20, 30, 40, 50, and 60 min. The samples were subjected to an assay for urea (Urea Colorimetric Assay Kit (Diacetyl Oxime Method), Elabscience, Wuhan, China) according to the kit procedure. Then, the inhibition rate was calculated as described previously [22].

### 2.8. Measurement of the Diuretic Effect in Rats and Mice

In a single-dose administration experiment, male wild-type (WT) mice, UT-B^−/−^ mice, UT-A1^−/−^ mice, or SD rats were adapted in metabolic cages for 3 d. Food and water were provided ad libitum throughout the experiment. Prior to administration, the bladder of each animal was emptied through gentle abdominal massage, and urine was collected from the metabolic cages every 2 h. Compound E3, suspended in 0.5% CMC-Na at an appropriate concentration, was administered intragastric gavage to the mice or rats (0.16, 0.8, 4, or 20 mg/kg). Vehicle control comprised 0.5% (*w*/*v*) CMC-Na. Urinary parameters were determined through the following: volume: Gravimetric analysis (1 g/mL density assumption); osmolality: freezing-point osmometry (Micro-osmometer, Fisker Associates, Norwood, MA, USA); and urea: diacetyl oxime colorimetry (QuantiChrom^®^ kit, Elabscience, Wuhan, China). In long-term (7 d) diuretic activity experiments, male WT mice, UT-B^−/−^ mice, UT-A1^−/−^ mice, and SD rats were acclimatized in metabolic cages (Ugo Basile, Comerio, VA, Italy) for 3 d. Food and water were provided ad libitum throughout the experiment. E3 was suspended in 0.5% CMC-Na and administered at a dose of 4 or 20 mg/kg by intragastric gavage. Urine was collected by metabolic cages every 24 h. Body weight was measured daily. At 4 h after the final administration, a blood sample was obtained by heart puncture. Inner medulla and outer medulla tissue homogenates were prepared, and the supernatant after centrifugation was analyzed for solute concentration and osmolality. Urinary osmolality and urea concentration were measured as above. Serum Na^+^, K^+^, and Cl^−^ were measured in a clinical chemistry laboratory, while serum creatinine, ALT, and AST were measured through specific reagent kits (NJJC Bio, Nanjing, China).

### 2.9. Rat Hyponatremia Model Caused by SIADH

The protocol for dDAVP-induced hyponatremia was adapted from a previous study [27], with tolvaptan chosen as the positive control. Preliminary experiments determined the dose of dDAVP (0.25 ng/h). Under isoflurane anesthesia, a 0.5 μg/mL dDAVP solution was injected subcutaneously using an osmotic minipump (ALZET 2002, Cupertino, CA, USA; 200 μL, low rate of 0.5 μL/h for 7 d). Rats were housed individually in metabolic cages to collect urine. Before using the pump, they were given 62.5 mL/d 1.0 kcal/mL of liquid food. After pump implantation, the rats received 40 mL of 2.1 kcal/mL of liquid feed daily. The rats were divided into five groups: control group (sham operation), model group (0.5% poloxamer, solvent), 4 mg/kg tolvaptan group, 4 mg/kg E3 group, and 20 mg/kg E3 group. E3 was administered orally every 8 h, while tolvaptan was given intragastrically once daily. The schedule of the above experiment arrangement is shown in Figure 4A. At the end of the experiment, the rats were sacrificed.

### 2.10. Metabolic Stability Assays

Plasma from mice, rats, rabbits, and humans was obtained from blood after centrifugation at 3000 r/min for 10 min. Simulated gastric fluid (SGF, pH = 1, containing pepsin) and simulated intestinal fluid (SIF, pH = 6.8, containing trypsin) were configured previously in the experiments [28,29]. SD rats and mice were collected, and after removing the mucus layer from the small intestine and colon, 20 times the volume of liquid bacterial culture medium was added. An intestinal microbiota suspension was obtained after anaerobic cultivation on a shaking table at 37 °C for 12 h. In the aforementioned systems, E3 was added at a final concentration of 1 μmol/L at 37 °C for different incubation times (0, 10, 20, 30, 60, 120, and 240 min). In liver metabolic stability assays, E3 was incubated with liver microsomes or liver homogenate from mice or rats for 60 min at 37 °C. The stop solution, 5 ng/mL tolbutamide (internal standard, IS) in acetonitrile, was added to stop the metabolic process. Post-centrifugation (18,000× *g*, 10 min, 4 °C), supernatants were subjected to liquid chromatography–tandem mass spectrometry (LC–MS/MS). Analyte-to-IS peak area ratios quantified residual percentages via Equation (2):

Remaining% = (E3_peak area_/IS_peak area_ at different time)/(E3_peak area_/IS_peak area_ at 0 min) × 100%. (2)

The hepatic clearance (CL_hep_) values were calculated using the amount of microsomal protein (mice: 45 mg/g liver; rat: 45 mg/g liver) and liver weight (mice: 50 g liver/kg; rat: 40 g liver/kg).

### 2.11. Pharmacokinetic Study in Rats

Male SD rats were allowed free access to food in a controlled environment of 22 ± 2 °C, with a humidity level of 55% ± 5%, and maintained under a 12 h light/dark cycle. In the oral administration groups, E3 was delivered in a 0.5% CMC-Na solution at a dosage of 4 mg/kg body weight. Blood samples were collected by the orbital venous plexus and kept on ice at the following time points: 0.083, 0.167, 0.25, 0.5, 1, 2, 4, 6, 8, 12, and 24 h. E3 formulations (DMSO:PEG400:saline = 10:30:60, *v*/*v*) were administered via tail vein (1 mg/kg). Serial blood sampling (0.033, 0.083, 0.167, 0.333, 0.5, 1, 2, 4, 8, 12, and 24 h post-dose) yielded plasma via centrifugation (3000 r/min, 10 min, 4 °C), stored at −80 °C until PK analysis using DAS 3.2.8 (noncompartmental analysis model). The oral bioavailability (F) was calculated as in Equation (3):

F = (AUC_p.o._ × Dose_i.v._)/(AUC_i.v._ × Dose_p.o._) × 100% (3)

### 2.12. Tissue Distribution Study

Male SD rats were randomly divided into four groups and received a single oral dose of E3 at 4 mg/kg. Blood and tissue samples (i.e., heart, liver, kidney, spleen, lung, brain, testis, skin, muscle, leg bone, stomach, small intestine, and colon) were collected at 0.5 h, 2 h, 6 h, and 24 h after dosing. Tissues were perfused with cold saline, blotted, weighed, and homogenized (10 vol/wt ice-cold H_2_O; T10 homogenizer, IKA, Staufen, Germany). The rats’ right shin bone was decalcified in methanol (4 °C, 12 h). Processed samples (−80 °C storage) underwent LC-MS/MS analysis as described.

### 2.13. Sample Preparation and LC-MS/MS Method

The analyte E3 and IS were dissolved in DMSO to generate the stock solutions. The working calibration of E3 was gradient-diluted with MeOH/H_2_O (1:1, *v*/*v*). Three microliters of working solutions were spiked with 27 μL of the blank rat plasma to establish the calibration standards. The final concentrations of the standard samples were 1, 2, 5, 10, 50, 250, 500, and 1000 ng/mL. Quality control samples (3/100/800 ng/mL) were prepared using identical protein precipitation protocols. Briefly, 30 μL calibrators and biological matrices were mixed with 450 μL stop solution, vortexed (5 min), and centrifuged (18,000× *g*, 10 min, 4 °C). Processed supernatants (2 μL injection volume) were subjected to LC-MS/MS analysis.

The analysis was conducted by an Xevo TQ-S Cronos Triple Quadrupole Mass Spectrometer coupled with an ACQUITY UPLC (Waters Corp, Milford, MA, USA). The chromatographic separation was conducted in an ACQUITY UPLC BEH C18 1.7 µm column (Waters, Milford, MA, USA) at 40 °C with a flow rate of 0.4 mL/min. The mobile phases were 0.1% (*v*/*v*) formic acid in water (A) and pure ACN (B). The gradient elution program started with 20% of B, which was maintained until 0.5 min, then phase B was increased to 90% in 1.5 min, and further increased to 100% in 1 min, and finally decreased to 30% of B in 1 min. An electrospray ionization (ESI) source with multiple–reaction monitoring (MRM) in positive–ion mode was employed to quantify the analytes with the transitions of *m*/*z* 385.11 > 244.19 and *m*/*z* 271.136 > 91.01 for E3 and IS, respectively. The optimized cone voltage is 10 V and 24 V for E3 and IS, respectively. The collision energy is 12 V and 32 V for E3 and IS, respectively. MassLynx v4.2 (Waters Corp, Milford, MA, USA) was used for data acquisition and analysis.

### 2.14. Toxicological Test Methods

#### 2.14.1. Acute Oral Toxicity Test

The acute oral toxicity test was performed according to “Guidance on Single Dose Toxicity Study for Pharmaceuticals” (2014), “Guidance for industry: single dose acute toxicity testing for pharmaceuticals” (1996). The initial dose for preliminary trials was established at 5000 mg/kg. A total of three female and three male mice were utilized (one of each sex for preliminary experiments and two for subsequent experiments). Following a 12 h fasting period, one male and one female mouse were selected and administered E3. After 24 h of confirming survival and the absence of abnormal behavior, the remaining four animals were given the same dose. The control group was given solvent (0.5% CMC-Na). Observations of all animals on the day of dosing (d 0) and in the days following were recorded, including general conditions (signs of toxicity, onset of symptoms, recovery period), changes in body weight, and mortality. After 14 d, blood and vital organ tissues were collected, and serum biochemical indicators as well as histopathological examinations were conducted.

#### 2.14.2. Subacute Oral Toxicity Test

The subacute oral toxicity test was performed according to “Guidance on Repeated Dose Toxicity Study for Pharmaceuticals” (2014). In the SIADH model, we found that 4 mg/kg of E3 produced good therapeutic effects, so we chose a dose of 1000 mg/kg, which is greater than 250 times the minimum effective dose, for the subacute toxicity test. Eighteen male mice were evenly divided into two groups. Mice of the control group were orally administered with solvent (0.5% CMC-Na). Another group was orally administered with E3 1000 mg/kg. The solvent or E3 was administered once a day for 30 continuous days and withdrawn for 14 d for recovery. After the 30-day treatment, 12 mice (6/group) were chosen at random and sacrificed for necropsy. The remaining 6 mice (3/group) were observed for 14 d after the cessation of treatment and sacrificed for necropsy after the recovery period.

During the study, the clinical symptoms, morbidity, and mortality of mice were observed twice a day at the side of the cage. Changes in appearance, behavior, appetite, gait, secretions, and excretions of the rats were meticulously documented. The body weights were measured before the start of the experiment, and the weights were measured every 2 d thereafter. Echocardiography was performed and evaluated every 10 d. Under anesthesia, blood samples were taken before the autopsy for hematological and clinical biochemistry analysis by an automated hematology analyzer (HEMAVET 950FS, Drew Scientific, TX, USA) and an automated biochemical analyzer (BS-180, Mindray Diagnostics, Shenzhen, China). The following organs were weighed: heart, liver, kidney, spleen, lung, thymus gland, testis, and brain. Furthermore, the corresponding organ/body weight ratio was computed. A comprehensive histopathological analysis was performed on vital tissues and organs.

#### 2.14.3. Assessment in Cardiotoxicity by hERG K^+^ Channel

CHO-hERG cells were maintained in F12 medium (Gibco, Thermo Fisher Scientific, Waltham, MA, USA) containing 10% FBS and 0.5 mg/mL Geneticin (Invitrogen, Thermo Fisher Scientific, Waltham, MA, USA) under standard culture conditions (37 °C, 5% CO_2_). Cells were seeded 2 days prior to reaching 70% confluency, then washed with PBS and detached using 5 mL Detachin (Genlantis, San Diego, CA, USA) at 37 °C for 2~3 min. Cell suspensions (2 × 10^6^ cells/mL in F12) were prepared and equilibrated for 20 min in the QStir station of the QPatch-16 system (Sophion Bioscience, Copenhagen, Denmark) before electrophysiological recordings.

hERG channel tail currents were assessed using the QPatch automated platform. Recording solutions contained (mM): Internal: KCl 120, CaCl_2_ 5.374, MgCl_2_ 1.75, KOH 31.25, EGTA 10, HEPES 10, Na_2_ATP 4 (pH 7.2); External: NaCl 145, KCl 4, MgCl_2_ 1, CaCl_2_ 2, HEPES 10, glucose 10 (pH 7.4)

Cells were voltage-clamped at −80 mV with series resistance monitored during −100 mV hyperpolarization. The stimulation protocol included: (1) 200 ms baseline at −50 mV; (2) 2s depolarization to +20 mV; and (3) tail current recording at −50 mV. Signals were acquired at 2 kHz through the integrated Bessel filter. Concentration-response relationships were established by sequential compound applications bracketed with saline controls for normalization.

#### 2.14.4. Mouse Sperm Malformation Assay

Male mice were randomly divided into three groups: control group (0.5% CMC-Na), E3 2000 mg/kg group, and positive group (50 mg/kg cyclophosphamide (CP), administered via intraperitoneal injection). E3 and the control group were administered once daily by intragastric gavage. All mice received treatment for 5 consecutive days. After the initial dosing period, which lasted 35 d, the mice were euthanized, and evaluation of sperm parameters was performed as follows:

Caudal epididymides were dissected into fragments in physiological saline and incubated (37 °C, 5% CO_2_, 10 min). Spermatozoa were isolated through nylon mesh filtration. Semen parameters were quantified using computer-assisted semen analysis (CASA). Eosin-stained smears (20 μL sperm suspension) were analyzed to examine sperm morphology. A total of 1000 sperm per sample were observed to identify the abnormality. Sperm shape abnormalities, such as banana, headless, without a hook, amorphous, double-tailed, and fat head, were identified and counted. In addition, the testis was reserved for morphological analysis. The spermatogenesis was graded from 1 to 10 using the Johnsen score [30,31,32].

### 2.15. Histology

Multiple tissues and organs were fixed with 4% paraformaldehyde and embedded in paraffin. Five μm paraffin sections were cut and stained with hematoxylin and eosin (H&E).

### 2.16. Western Blot Analysis

Inner medulla tissues were homogenized in ice-cold RIPA buffer supplemented with protease/phosphatase inhibitors (Roche, Basel, Switzerland). Following centrifugation (12,000× *g*, 15 min, 4 °C), supernatants were subjected to BCA quantification (Thermo Scientific, Waltham, MA, USA). Protein lysates underwent SDS-PAGE separation and electrotransfer to PVDF membranes (Amersham Biosciences, Boston, MA, USA). After 2 h blocking with 5% skimmed milk, membranes were sequentially processed with: (1) TBST washes (20 mM Tris-HCl, 137 mM NaCl, 0.1% Tween-20, pH 7.4); (2) primary antibodies (UT-A1/A2/A3, UT-B, AQP2/3, β-actin; 4 °C, 12 h); and (3) HRP-conjugated secondary antibodies (anti-mouse/rabbit IgG; RT, 2 h). Chemiluminescent detection employed the ECL reagent (Meilunbio, Dalian, China) on a Syngene GeneGnome XRQ system, with band quantification via ImageJ (v1.53k).

### 2.17. Statistical Analysis

Data analysis was performed using GraphPad Prism 8 with continuous variables expressed as mean ± SEM. Intergroup differences were analyzed by Student’s *t*-test, while multigroup comparisons utilized one-way ANOVA with Tukey’s correction. A *p*-value < 0.05 was considered statistically significant.

## 3. Results

### 3.1. E3 Was Identified from Structurally Optimized Diarylamides

In conjunction with our prior research, we have determined that the acetylfuran fragment and the amide linker are key structural elements that enhance the bioactivity of the diarylamide compounds [23]. To enhance water solubility, we initially modified compound 25a by introducing ethylpiperazine (E1) and N-(2-(dimethylamino)ethyl)formamide (E2) (Table 1). The inhibitory activities of these derivatives against rat and mouse UT-B were comparable to those of 25a, indicating that the amide bond adjacent to the benzene ring is not a critical functional group. Previous studies have demonstrated that sulfonamides can enhance compound activity [23]; thus, we further designed E3, E4, and E5 by incorporating arylsulfonamides at different positions on the benzene ring. The activity assays revealed that substitution at the meta position yielded the highest activity, followed by the para position, while substitution at the ortho position resulted in the lowest activity. In benzene derivatives, the terms ortho, meta, and para describe the relative positions of two substituents on the benzene ring [33,34,35]. Ortho indicates adjacent carbons (positions 1,2), meta indicates a one-carbon separation (positions 1,3), and para indicates opposite positions (positions 1,4).

Additionally, to mitigate the potential cytotoxicity associated with sulfonamide acidity, we introduced a hydroxyl group at the ortho position of the sulfonamide in compound E5, with the intention that this hydroxyl group would form a hydrogen bond with the sulfonamide amino group, thereby reducing the ionization of the sulfonamide and leading to the design of compound E6. Concurrently, to prevent the formation of phenylenediamine metabolites, we swapped the sulfonyl and amine groups, resulting in compound E7. Activity assays indicated that compound E7 also exhibited significant UT-B inhibitory activity. Building on E7, we introduced a water-soluble morpholine group at the para position of the benzene ring and designed connecting arms of varying lengths (E8 and E9). The results indicated that the introduction of the morpholine ring resulted in a slight decrease in compound activity; however, it remained superior to the lead compound 25a.

Activity studies have demonstrated that the combination of benzene sulfonamide and acetyl furan (E3~E5) significantly enhances the biological activity of the compound. Notably, the activity of benzene sulfonamide at the meta position is the highest, followed by the para position, while the ortho position exhibits the lowest activity. These enhancements prompted the identification of several candidates, with E3 displaying the most potent inhibitory activity. Consequently, E3 was chosen for further extensive preclinical studies. The chemical structure of compound E3, named 5-acetyl-N-(3-(phenylsulfonamido)phenyl)furan-2-carboxamide, is shown in Figure 1A, indicating the structural optimization process from 25a to E3.

### 3.2. E3 Dose Dependently Inhibited UT-A1 and UT-B

The IC_50_ values of E3 for UT-B-promoted urea transport, as determined by the red blood cell lysis assay, were found to be 47.7 nM in mice, 7.6 nM in rats, 17.7 nM in rabbits, and 25 nM in humans, respectively (Figure 1B–D), indicating a greater inhibitory activity than the compound 25a (480 nM in mice and 140 nM in rats). The maximum inhibition rates of E3 in mouse, rat, rabbit, and human cells approached 100%. As a control, the erythrocyte lysis rate in UT-B^−/−^ mice was approximately 100%, with or without E3 incubation, reflecting the absence of UT-B in the erythrocyte membrane (Figure 1B).

Stopped-flow assays were conducted to assess the inhibitory effect of compounds on UT-B. Following the mixing of red blood cells with a high-urea solution, the cells rapidly shrink due to water loss mediated by aquaporin 1 (AQP1), followed by an increase in cell size due to UT-B-mediated urea influx and concurrent water influx via AQP1 [22]. The changes in cell volume during this process can be monitored through variations in scattered light. After incubation with E3 and subsequent exposure to a high urea solution, it was observed that E3 could dose-dependently inhibit the function of UT-B on the surface of erythrocytes in transporting urea into the cells (Figure 1E). In the efflux experiment, erythrocytes were incubated in a high-urea environment and then mixed with isotonic PBS. It was found that E3 also inhibited UT-B-mediated urea efflux in a dose-dependent manner (Figure 1F). Furthermore, the inhibition was reversible, as demonstrated by exposing erythrocytes to E3 at 1 μM followed by washout with PBS (Figure 1G).

We used MDCK cell lines stably expressing rat UT-B or UT-A1 to evaluate the selectivity of E3 towards UT-A1 and UT-B. The experimental results showed that E3 significantly inhibited both UT-A1 and UT-B-mediated urea permeabilities, with a greater inhibitory effect against UT-A1 (IC_50_ = 18 nM) than UT-B (IC_50_ = 58 nM) (Figure 1H).

### 3.3. E3 Exerted Diuretic Effect Mainly by Inhibiting UT-A1

**Figure 2:**
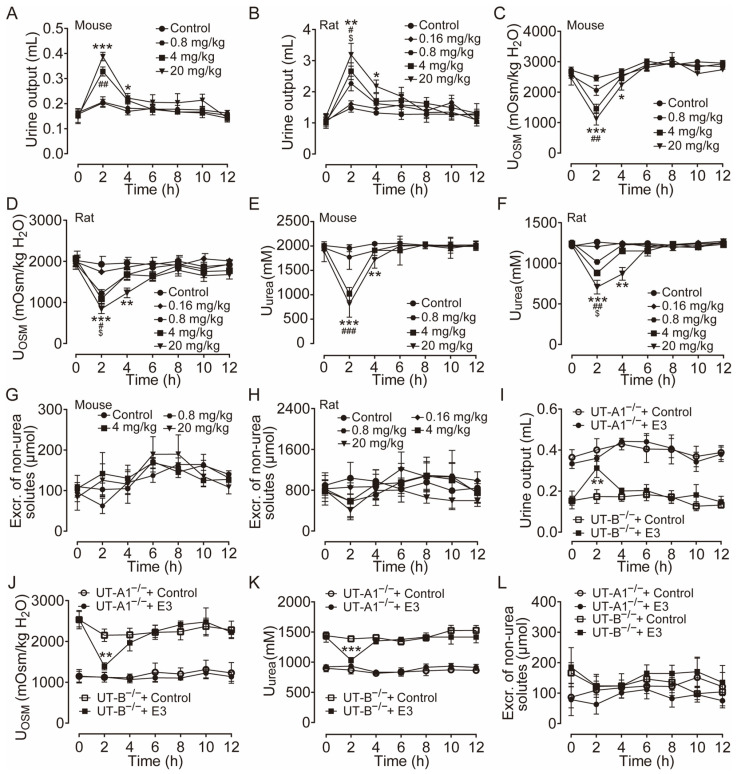
Diuretic effects of single intragastric administration of E3 in mice and rats. (**A**) Urine output of mice. (**B**) Urine output of rats. (**C**) Urinary osmolality of mice. (**D**) Urinary osmolality of rats. (**E**) Urinary urea concentration of mice. (**F**) Urinary urea concentration of rats. (**G**) Excretion of non-urea solutes of mice. (**H**) Excretion of non-urea solutes of rats. (**I**) Urine output of UT-A1^−/−^ and UT-B^−/−^ mice. (**J**) Urinary osmolality of UT-A1^−/−^ and UT-B^−/−^ mice. (**K**) Urinary urea concentration of UT-A1^−/−^ and UT-B^−/−^ mice. (**L**) Excretion of non-urea solutes of UT-A1^−/−^ and UT-B^−/−^ mice. Data are presented as mean ± SEM (n = 6). * *p* < 0.05, ** *p* < 0.01 and *** *p* < 0.001, E3 20 mg/kg vs. Ctr. # *p* < 0.05, ## *p* < 0.01 and ### *p* < 0.001, E3 4 mg/kg vs. Ctr. $ *p* < 0.05, E3 0.8 mg/kg vs. Ctr.

The diuretic effect of E3 was determined in mice and rats using metabolic cages. Following the 3 d acclimatization period in the metabolic cages, the animals were provided a standard diet and had free access to drinking water. The mice and rats received intragastric gavage varying doses of E3 or a solvent control, with urine being collected every 2 h both before and after administration. Urine output significantly increased in both mice (Figure 2A) and rats (Figure 2B) in a dose-dependent manner after E3 intragastric gavage compared to the vehicle control. Concurrently, urinary osmolality (Figure 2C,D) and urea (Figure 2E,F) decreased in the same experiment. The excretion of non-urea solutes was not significantly different between E3 groups and the control group in both mice and rats (Figure 2G,H), indicating that E3 does not influence electrolyte excretion. The inhibitory selectivity of E3 on UT-A1 and UT-B was assessed in UT-A1^−/−^ mice and UT-B^−/−^ mice administered 20 mg/kg E3. In UT-B^−/−^ mice, urine output markedly increased 2 h post-E3 administration (Figure 2I), the urinary osmolality (Figure 2J) and urea (Figure 2K) decreased correspondingly. However, there was no significant change in urine output, urinary osmolality, and urea concentration in UT-A1^−/−^ mice. These data suggest that E3 exerts a significant diuretic effect by mainly inhibiting UT-A1. The excretion of non-urea solutes (Figure 2L) was not significantly changed in both colonies.

### 3.4. Diuretic Activity of Long-Term Administration of E3 In Vivo

**Figure 3:**
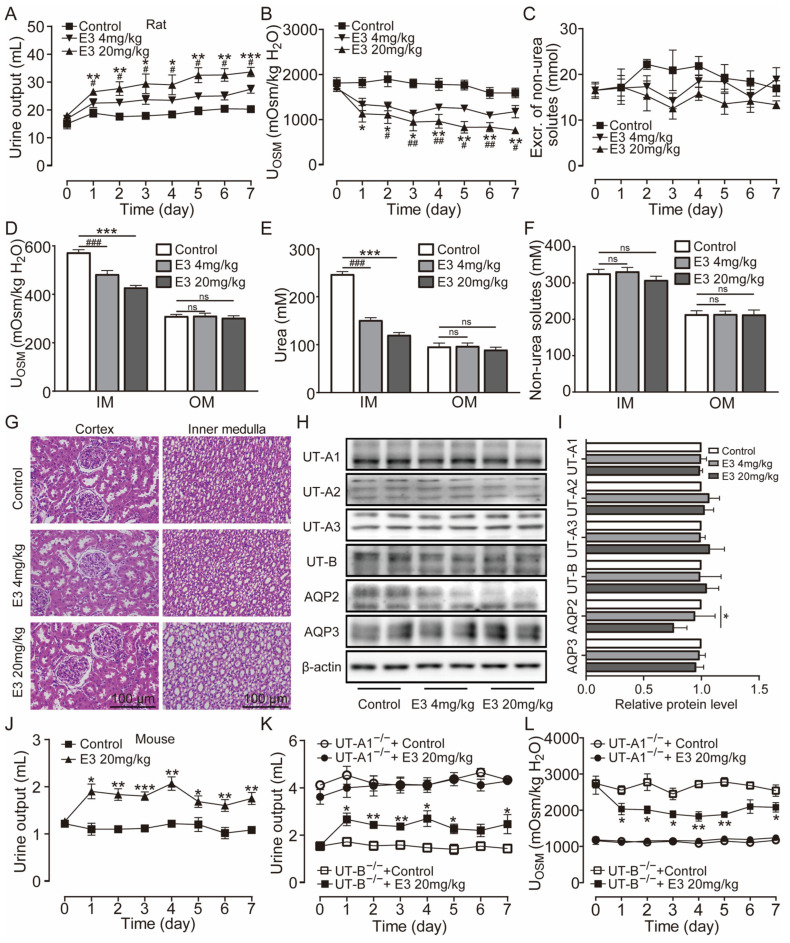
Long-term diuretic effect of E3 in mice and rats. (**A**) Urine output, (**B**) urine osmolality, (**C**) excretion of non-urea solutes, (**D**) osmolality in renal medulla tissues, (**E**) urea concentration in renal medulla tissues, (**F**) concentration of non-urea solutes in renal medulla tissues. (**G**) H&E-stained kidney tissue sections. (**H**) Representative Western blotting of UTs and AQPs in renal medulla homogenate. (**I**) The relative protein expression levels of rats receiving consecutive administrations of E3 at 4 and 20 mg/kg, data are presented as mean ± SEM (n = 4). (**J**) Urine output of mice. (**K**) Urine output of UT-A1^−/−^ and UT-B^−/−^ mice. (**L**) Urinary osmolality of UT-A1^−/−^ and UT-B^−/−^ mice. OM, outer medulla; IM, inner medulla. Data are presented as mean ± SEM (n = 6). * *p* < 0.05, ** *p* < 0.01 and *** *p* < 0.001, E3 20 mg/kg vs. Ctr. # *p* < 0.05, ## *p* < 0.01 and ### *p* < 0.001, E3 4 mg/kg vs. Ctr. ns, no significance.

Clinically, diuretic drugs are typically employed for long-term treatment. Therefore, we further studied the pharmacological characteristics of E3 for long-term diuretic effects. UT-A1^−/−^ mice, UT-B^−/−^ mice, and rats received intragastric administration of E3 for 7 d. Following administration of 4 mg/kg and 20 mg/kg E3 to rats, urine output continuously increased (Figure 3A), urine osmolality significantly decreased (Figure 3B), and excretion of non-urea solutes did not change (Figure 3C). Compared to the control group, the osmolality (Figure 3D) and urea concentration (Figure 3E) significantly decreased, while non-urea solutes did not change (Figure 3F) in the inner medullary tissue of E3-treated rats. However, there were no differences in outer medullary osmolality, urea concentration, and non-urea solute concentration between E3-treated rats and control rats (Figure 3D–F). These results indicate that E3 exerts a diuretic effect by blocking the intrarenal urea recycling without interfering with the excretion of Na^+^, K^+^, and Cl^−^. In addition, after 7 d of E3 treatment in rats, there was no significant difference in body weight, organ indexes, or various blood biochemical parameters (including serum Na^+^, K^+^, and Cl^−^) compared to the control rats (Table S1). H&E staining showed that continuous administration of E3 did not induce morphological abnormalities in the cortex and medulla of rat kidneys, except dilatation of the collecting duct in medullary tissue due to polyuria (Figure 3G).

Western blot analysis of UTs and aquaporins (AQPs) in the medullary tissue of rats revealed that E3 did not significantly alter the expression levels of UT-A1, UT-A2, UT-A3, and UT-B and AQP3 (Figure 3H,I). The AQP2 expression in 20 mg/kg E3-treated rats was lower than control rats, which may be due to long-term polyuria and low urinary osmolality that decreased AQP2 protein trafficking to the apical membrane. All these data suggest that E3 had a diuretic effect by selectively inhibiting UT-A1, without disturbing electrolyte balance, normal metabolism, or renal function.

Subsequent experiments were conducted in mice. Following oral administration of 20 mg/kg E3, the urine output significantly increased (Figure 3J), urine osmolality (Figure S2A), and urea concentration were significantly decreased (Figure S2B). There was no significant difference in the excretion of non-urea solutes (Figure S2C). After long-term treatment of E3, urine output increased (Figure 3K) and urine osmolality decreased (Figure 3L) in UT-B^−/−^ mice, whereas there was no significant diuretic effect in UT-A1^−/−^ mice, which suggests that the diuretic effect of E3 is mainly based on UT-A1 inhibition.

### 3.5. E3 Had Therapeutic Effects on the Hyponatremia of SIADH

**Figure 4:**
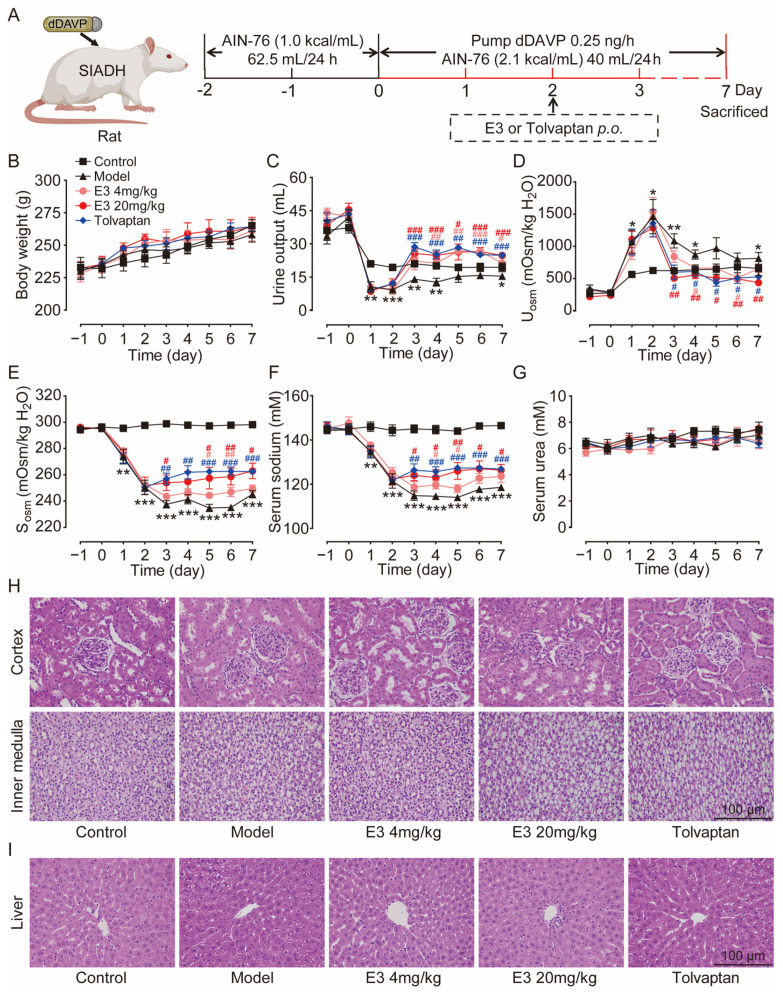
The effect of E3 on the SIADH model. (**A**) Experimental scheme for establishing the SIADH model. (**B**) Body weight. (**C**) Urine output. (**D**) Urine osmolality. (**E**) Serum osmolality. (**F**) Serum sodium concentration. (**G**) Serum urea concentration. (**H**) Representative images of kidney tissue sections. (**I**) Representative images of liver tissue sections. Data are presented as mean ± SEM (n = 6). * *p* < 0.05, ** *p* < 0.01 and *** *p* < 0.001, model vs. Ctr. # *p* < 0.05 and ## *p* < 0.01, ### *p* < 0.001 E3 4 mg/kg vs. model. # *p* < 0.05, ## *p* < 0.01, ### *p* < 0.001, E3 20 mg/kg vs. model. # *p* < 0.05, ## *p* < 0.01, ### *p* < 0.001, tolvaptan vs. model.

The therapeutic effect of E3 on hyponatremia was subsequently evaluated in a rat model with the SIADH, as illustrated in Figure 4A. Throughout the experiment, all rats showed moderate weight gain (Figure 4B). Following the infusion of dDAVP via a pump, the urine output in the model rats was significantly reduced (Figure 4C), while urine osmolality was increased in comparison to the control rats (Figure 4D). Concurrently, serum osmolality and sodium levels decreased in the model rats (Figure 4E,F), confirming the successful establishment of the SIADH hyponatremia model. After the treatment of E3 or tolvaptan (the positive control), the hyponatremia in SIADH rats was significantly alleviated, with significantly increased serum osmolality and sodium concentration compared to the model rats. There was no significant difference in blood urea levels between the experimental groups before and after treatment (Figure 4G). In addition, H&E staining also showed normal tissue structures in the kidney, except for dilatation of collecting ducts, and liver of E3 or tolvaptan-treated rats (Figure 4H,I). These results indicate that E3 alleviates hyponatremia through its diuretic mechanism.

### 3.6. E3 Had Good Metabolic Stability In Vitro and Pharmacokinetic Characteristics In Vivo

**Figure 5:**
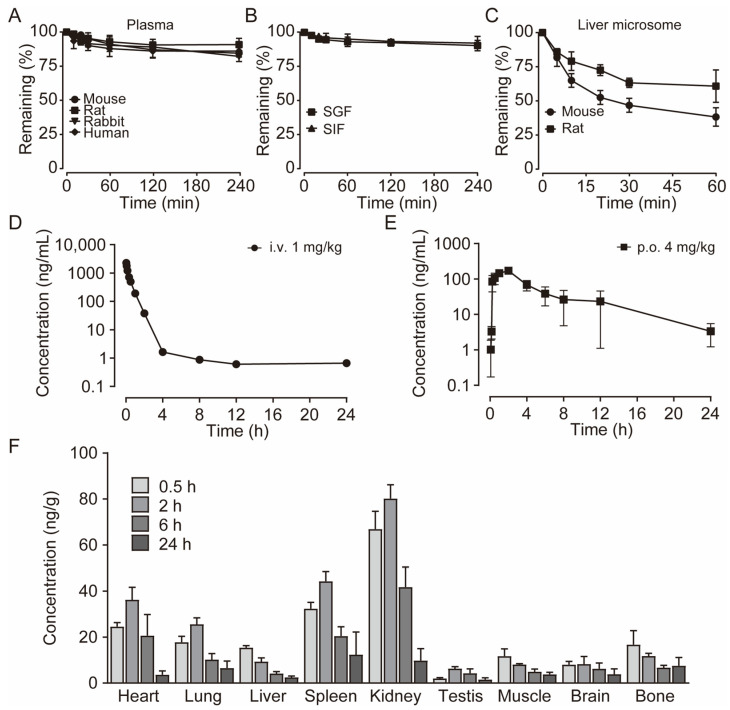
The pharmacokinetic profiles of E3. (**A**) Stability of E3 in the plasma of various species. (**B**) Stability of E3 in simulated gastric fluid (SGF) and simulated intestinal fluid (SIF). (**C**) Stability of E3 in mouse and rat liver microsomes. Data are presented as mean ± SD (n = 3). (**D**) Mean plasma concentration of E3 in SD rats after a single *i.v.* dose at 1 mg/kg body weight. (**E**) Single *p.o.* dose at 4 mg/kg body weight. (**F**) Tissue distribution of E3 in SD rats after a single oral dose at 4 mg/kg. Data are presented as mean ± SEM (n = 5).

To characterize the metabolism of E3, we assessed its metabolic stability in blood and plasma, liver microsomes, and liver homogenate. After incubating E3 in the plasma of various species at 37 °C for 4 h, we found that over 81% of E3 remained, indicating that E3 exhibits significant stability in the plasma of these species (Figure 5A). The remaining amount after blood incubation has slightly decreased (Table S2). Oral administration offers better patient compliance and facilitates long-term use. Consequently, we examined the stability of compound E3 in simulated gastric fluid (SGF) at pH 1.0 and simulated intestinal fluid (SIF) at pH 6.8 (Figure 5B). More than 90% of E3 remained after 4 h. In addition, after incubating with intestinal microbiota (colon and small intestine), over 82% of E3 persisted after 4 h (Table S2). Following 60 min of incubation in mouse and rat liver microsomes and liver homogenate, the remaining amounts were 36.8%, 39.8%, 61.1%, and 69.0%, respectively, suggesting varying degrees of metabolism and notable species differences (Figure 5C) (Table S2). These results suggest that liver metabolism may be the primary elimination pathway for E3, while its metabolic stability in the gastrointestinal tract is notably high.

The validated LC–MS/MS method was utilized in the pharmacokinetic study of E3 in rats following a single intravenous administration at a dose of 1 mg/kg and single oral dose of 4 mg/kg. The mean plasma concentration–time profiles for both the single intravenous dose groups and the single oral dose are presented (Figure 5D,E). The pharmacokinetic parameters derived from noncompartmental analysis (NCA) are summarized in Table 2. Notably, the concentration of E3 in rat plasma reached its maximum concentration (*C_max_*) within 2 h after oral administration, indicating moderate oral absorption in rats. The half-life of E3 in rats following single oral doses was approximately 4.9 h, while the mean residence time (MRT) was around 6.1 h. The bioavailability was measured at 21.1%, which may be attributed to the limited water solubility of E3 and potential absorption saturation, warranting further investigation.

The tissue distribution experiment demonstrated the concentrations of E3 in plasma and various tissue samples at 0.5, 2, 6, and 24 h post a single oral administration of 4 mg/kg E3 in rats (Figure 5D). Post-administration, E3 exhibited widespread distribution across the rat tissues. Notably, the E3 concentrations were elevated in organs with high blood perfusion, such as the kidney, heart, lung, and spleen, with the kidney exhibiting the highest concentration (Figure 5D). Conversely, E3 showed relatively low distribution in the brain, testis, indicating that E3 has lower permeability to the blood–brain barrier (BBB) and blood–testis barrier (BTB). The drug concentration in most tissues peaked at 2 h post-administration, then was eliminated gradually.

### 3.7. Toxicity Assay Showed the Safety of E3

In the 14-day acute toxicity assay conducted on mice, all animals survived and exhibited normal behavior and good mental state, indicating LD_50_ > 5000 mg/kg (Figure S3A). During the test, the weight of the mice treated with E3 increased steadily, and the representative blood biochemical indicators were not statistically altered (Figure S3B–F). Furthermore, the organ indexes of E3-treated groups showed no significant difference compared to the control group (Table S3). H&E staining results indicated that the heart, kidney, liver, spleen, and brain of the mice were structurally intact, with clear outlines and no apparent abnormalities (Figure S3G).

**Figure 6:**
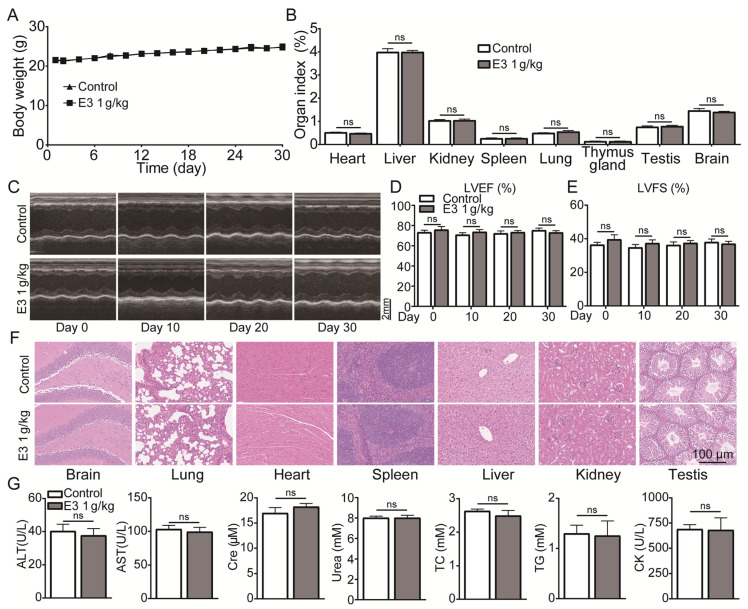
Subacute oral toxicity assay in mice. (**A**) Body weight. (**B**) Organ indexes. (**C**) Representative M-mode images at the parasternal long axis. (**D**) Left ventricular ejection fraction. (**E**) Left ventricular fractional shortening. (**F**) H&E staining of tissues from the brain, lung, heart, spleen, liver, kidney, and testis. (**G**) Blood biochemical indexes, including ALT, AST, Scr, urea, total cholesterol (TC), triglycerides (TG), and creatine kinase (CK). Data are presented as mean ± SEM (n = 9). ns, no significance.

During 30 days of continuous 1000 mg/kg E3 daily administration, there was no apparent abnormality in the appearance, behavior, secretions, and excretions of all treated mice. There was no significant change in body weight or organ indexes (Figure 6A,B). In addition, the results of cardiac ultrasound showed no significant abnormality in the left ventricular ejection fractions (LVEF) and left ventricular fractional shortening (LVSF) indicators of mice during and after the administration of E3 (Figure 6C–E). No histopathological toxic lesion related to the treatment with E3 was found in the brain, lung, heart, spleen, liver, kidney, or testis (Figure 6F). The analysis of blood samples showed that there were no significant changes in blood parameters and blood biochemical indexes between the E3-treated and control groups (Figure 6G) (Table S4). Observing mice during the recovery period, it was found that compared with the control group, all mice in the E3 group survived without any abnormal changes in body weight and organ indexes, indicating that E3 had no delayed toxicity (Figure S4).

Blockade of the hERG (human ether-a-go-go-go related gene) K^+^ channel and the consequent prolongation of the QT interval on the ECG have been considered the gold standard in non-clinical development studies aimed at supporting regulatory guidance (ICHS7) to predict the arrhythmogenic risk of drugs [36,37,38,39,40]. Therefore, we evaluated the effect of compound E3 on the hERG K^+^ channel. Encouragingly, E3 showed a low inhibitory effect on the hERG K^+^ channel with an IC_50_ value greater than 33.3 μM (Table S2), indicating low potential cardiac safety issues.

**Figure 7:**
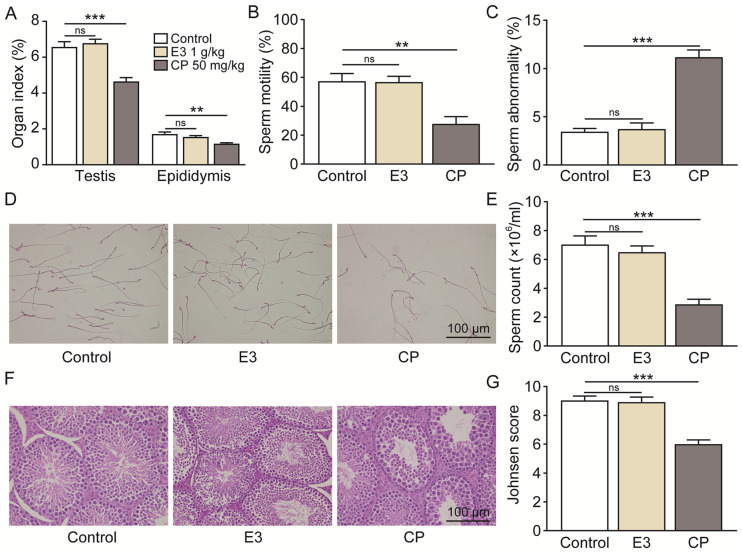
Male reproductive toxicity detection and safety analysis of E3. (**A**) Testicular and epididymal indexes. (**B**) Sperm motility. (**C**) Sperm abnormality. (**D**) The representative photographs of sperm stained with eosin. (**E**) Sperm count. (**F**) Representative images of testicular H&E staining. (**G**) Johnsen score of the seminiferous tubules. Data are presented as mean ± SEM (n = 6). ns, no significance. ** *p* < 0.01 and *** *p* < 0.001, vs. Ctr.

Mouse sperm malformation assays are a common type of genotoxicity assay [41]. On the 35th day following the oral administration of 2000 mg/kg/day of E3 for five consecutive days, no significant differences were observed in the testicular and epididymal indices (Figure 7A), sperm motility (Figure 7B), the percentage of sperms with abnormal morphology (Figure 7C), sperm counts (Figure 7D,E), or Johnsen score (Figure 7G) when compared to the control group. Histological examination using H&E staining revealed no significant differences in testicular tissue between the E3 mice and the control group (Figure 7F). In contrast, the reproductive toxic compound cyclophosphamide (CP) caused abnormalities in male reproductive indexes (Figure 7A–G).

## 4. Discussion

Our previous study found that the UT inhibitor diarylamides 1H and 25a had oral diuretic activity with the potential to be developed into novel diuretics [22,23]. However, these diarylamide compounds have some limitations that restrict their suitability for further development. Hence, the motivation of this study is to discover new diarylamides with strong diuretic activity and good druggability, and to develop them into candidate drugs.

In this study, we conducted a series of structural optimizations on the primary compound 25a. The 5-acetylfuran fragment and the benzenesulfonamide fragment can enhance UT inhibitory activity. Therefore, we spliced these two fragments using amide bonds to design compounds E3 to E5. Additionally, to address the potential cytotoxicity associated with the acidity of sulfonamide, we introduced a hydroxyl group into the ortho position of the amide, aiming for this hydroxyl group to form a hydrogen bond with the sulfonamide amino group, thereby reducing the ionization of the sulfonamide, which led to the design of compound E6. Furthermore, considering the potential toxic metabolites of phenylenediamines, we also designed compound E7. To improve the water solubility and molecular weight of the compounds, we incorporated ethoxymorpholine (E8) and propoxymorpholine (E9) into the sulfonamide benzene ring, resulting in a total of nine compounds.

The analysis of the structure-activity relationship indicates that the combination of benzene sulfonamide and acetyl furan (E3 and E5–E7) significantly enhances the UT inhibitory activity of the compounds. The IC_50_ in rat erythrocytes was <0.1 μmol/L, representing an increase in two orders of magnitude, with the activity ranking as meta- > para- > ortho-position. The ortho position of the phenyl group on the aniline side can accommodate small groups (E6), allowing for the subsequent introduction of additional groups to enhance the water solubility and metabolic stability of the compound. Moreover, based on E7, we introduced morpholine through a connecting arm (E8, E9). Although this modification slightly reduced activity, the inhibitory efficacy did not improve significantly compared to 25a. The water solubility of this class of compounds is significantly improved, potentially leading to higher bioavailability.

Subsequent in vitro and in vivo studies were conducted to assess the efficacy of the small-molecule inhibitor. Comparative inhibitory profiling of diarylamide compounds revealed that E3 demonstrated superior inhibitory activity against UT-B of multiple species. In addition, the Transwell assay found that E3 had stronger inhibitory activity against UT-A1 (IC_50_ = 18 nM) than UT-B (IC_50_ = 58 nM). Oral E3 administration to rats and mice exhibited stronger diuretic activity than 25a. Furthermore, single and long-term administration of E3 significantly increased urine output with a corresponding decreased urine osmolarity in UT-B knockout mice, but not in UT-A1 knockout mice, indicating that the diuretic effect of E3 is based on its highly selective UT-A1 inhibition.

Our previous studies confirmed that UT inhibitor 25a alleviated cirrhotic ascites and SIADH caused hyponatremia by exerting a diuretic effect [25,26]. Compared to 25a (100 mg/kg), E3 significantly improved SIADH hyponatremia at a lower dosage (20 mg/kg) due to its stronger diuretic activity. These data indicate that structurally optimized E3 has promising therapeutic potential to be developed into an oral diuretic.

The metabolic stability is a crucial aspect of new drug development [42,43,44,45]. Our study evaluated the pharmacokinetic profile of E3. In plasma metabolic stability tests across multiple species, E3 demonstrated high stability with over 80% of the remaining compound. As we aim to advance E3 as an oral diuretic, its robust stability in SGF, SIF, and intestinal microbiota gives more confidence in its further development. Furthermore, analysis of liver microsomes and homogenates indicated alternative metabolic pathways for E3, beyond phase I metabolism. Pharmacokinetic assessments in rats showed a *C_max_* of 170.3 ng/mL (443.0 nmol/L) for E3, surpassing the IC_50_ of UT-A1 (18 nmol/L), aligning with efficacy outcomes. Following intragastric gavage administration, plasma concentrations of E3 dropped to less than 2 ng/mL within 24 h, indicating rapid and complete elimination in rats. Notably, E3 exhibited prolonged *t*_max_ time (2 h vs. 0.25 h) and half-life (4.9 h vs. 2.86 h) compared to 25a, suggesting slow absorption and favorable long-term effects [24].

Tissue distribution results indicate that a high distribution of E3 in the kidney is a favorable characteristic for targeting renal UTs. Furthermore, E3 exhibits low permeability to both the blood–brain barrier and the blood–testis barrier, suggesting that the potential risks to the central nervous system and male reproductive system associated with E3 are likely negligible. Subsequent genotoxicity-related experiments have corroborated this finding.

Preclinical drug safety evaluation is a crucial step in the initial phase of new drug research and development [46,47,48,49]. The safety of E3 was assessed by acute, subacute, genotoxicity, and cardiotoxicity studies. In the acute toxicity experiment, mice were given a dose of 5000 mg/kg/day (>200 times the therapeutic dose). There were no animal deaths, significant adverse reactions, or tissue lesions during the 2-week period after E3 administration, indicating that E3 has no acute toxicity and has a broad safety margin. The results of the subacute toxicity test showed that there was no death, abnormal body weight, obvious abnormalities in vital organ indexes, or pathological change. Hematological and clinical biochemistry analysis showed relatively normal. Moreover, during the 2 week observation period after E3 administration for 30 d, no abnormal phenomena were found in the mice, indicating that E3 has no reversible toxic reaction and delayed toxicity, establishing 1000 mg/kg/day as the no observed adverse effect level (NOAEL). Cardiotoxicity hERG testing showed that E3 had a lower inhibitory effect on the hERG K^+^ channel, indicating potential safety. Regarding genotoxicity toxicity, results from sperm abnormality tests in mice at a dose of 2000 mg/kg/day for 5 consecutive days were all negative, indicating that E3 has a low risk of genotoxicity toxicity.

## 5. Conclusions

In this study, a new urea transporter inhibitor, E3, was obtained through structural modification of the diarylamide compound 25a. Compared to the lead compound, E3 exhibits high inhibitory activity against both UT-B and UT-A1, with greater selectivity towards UT-A1. E3 exhibits significant diuretic activity in rats and mice following oral administration without causing electrolyte imbalances, and improved SIADH at a lower dosage due to its stronger diuretic activity, confirming its efficacy in therapeutic potential. E3 significantly. Furthermore, E3 shows good metabolic stability in vitro and in vivo, with no apparent cytotoxicity, acute toxicity, subacute toxicity, genotoxicity, or cardiotoxicity observed. These preclinical data suggest that E3 possesses favorable druggability as a new diuretic. This study provides proof of concept that the diarylamide E3 has the potential to be developed into a novel diuretic for treating hyponatremia associated with volume expansion.

## Figures and Tables

**Table 1:** In vitro inhibitory activities of E1-E9 against UT-B (n = 3).

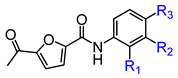					
Compound	R1	R2	R3	IC_50_ (μM) ^1^	
				Rat	Mouse
25a	H	H	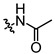	0.14	0.48
E1	H	H	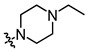	0.32	0.86
E2	H	H	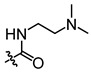	0.40	2.41
E3	H	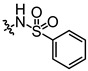	H	0.01	0.05
E4	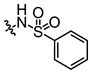	H	H	6.46	>10
E5	H	H	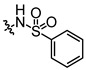	0.06	0.23
E6	H	OH	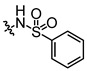	0.05	0.18
E7	H	H	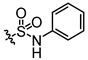	0.09	0.23
E8	H	H	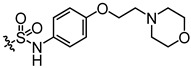	0.77	2.55
E9	H	H	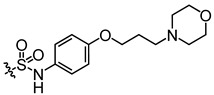	1.75	4.07

^1^ IC_50_ is tested with the erythrocyte lysis model. Data are means.

**Table 2:** Pharmacokinetic parameters of E3 in rats (n = 5).

Parameter	Unit	Single *i.v.* 1 mg/kg	Single *p.o.* 4 mg/kg
*t*_1/2_	h	1.9	4.9
*t*_max_	h	-	2.0
*C*_max_	ng/mL	2535.6	170.3
AUC_0-t_	ng/L × h	1115.1	919.4
AUC_0-∞_	ng/L × h	1116.8	943.17
MRT	h	1.1	6.1
V/F ^1^	L/kg	2.5	30.1
CL/F ^2^	L/kg/h	1.0	4.2
F	%	-	21.1

^1^ V and ^2^ CL for the *i.v.* group.

## Data Availability

Data are contained within the article.

## Supplementary Materials

The following supporting information can be downloaded at https://www.mdpi.com/article/10.3390/biomedicines13040992/s1, Figure S1: the reported type of UT inhibitors and the chemical structures of representative compounds; Figure S2: long-term diuretic effect of E3 in mice; Figure S3: acute oral toxicity assay in mice; Figure S4: subacute oral toxicity assay in recovery period. Table S1: body weight, organ indexes and blood chemistry in rats; Table S2: broader profiles of E3 in vitro; Table S3: organ indexes in mice in acute toxicity experiments; Table S4: routine blood examination of subacute toxicity assay in mice. NMR spectrum of the target compounds; HPLC chromatogram of compound E3.
