# Supplementary material for: Druggability Studies of Benzene Sulfonamide Substituted Diarylamide (E3) as a Novel Diuretic
Source: Biomedicines. 2025 Apr 18;13(4):992. doi: 10.3390/biomedicines13040992 (PMC12024912; doi:10.3390/biomedicines13040992)
Supplement: Supplementary file 1 [file biomedicines-13-00992-s001.zip › biomedicines-3567355-supplementary.pdf]

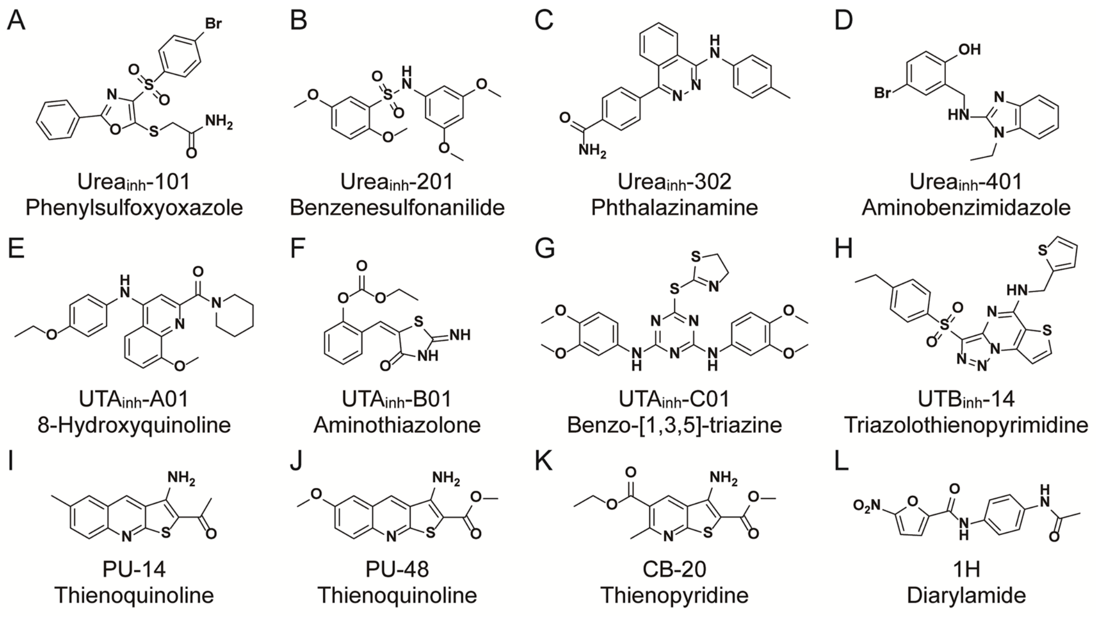

Figure S1. The reported type of UT inhibitors and the chemical structures of representative compounds.

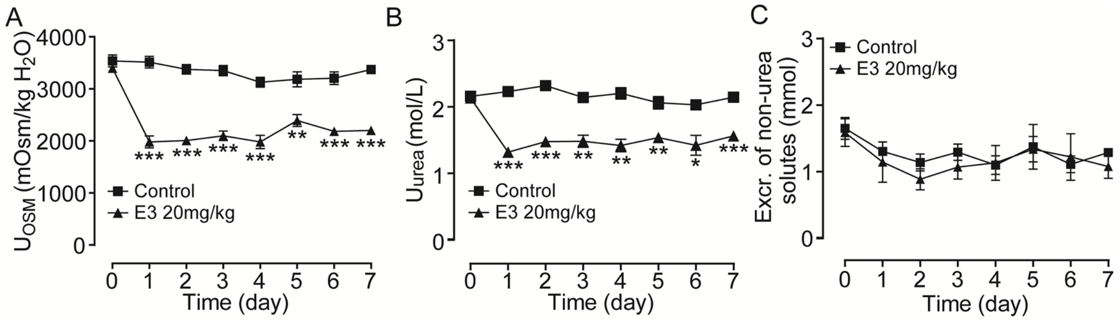

Figure S2. Long-term diuretic effect of E3 in mice. (A) Urine osmolality, (B) urine urea concentration, and (C) excretion of non-urea solutes of mice receiving consecutive administrations of E3 at 20 mg/kg. Data are presented as mean ± SEM (n = 6). *$p < 0.05$, **$p < 0.01$ and ***$p < 0.001$, E3 20 mg/kg vs. Ctr.

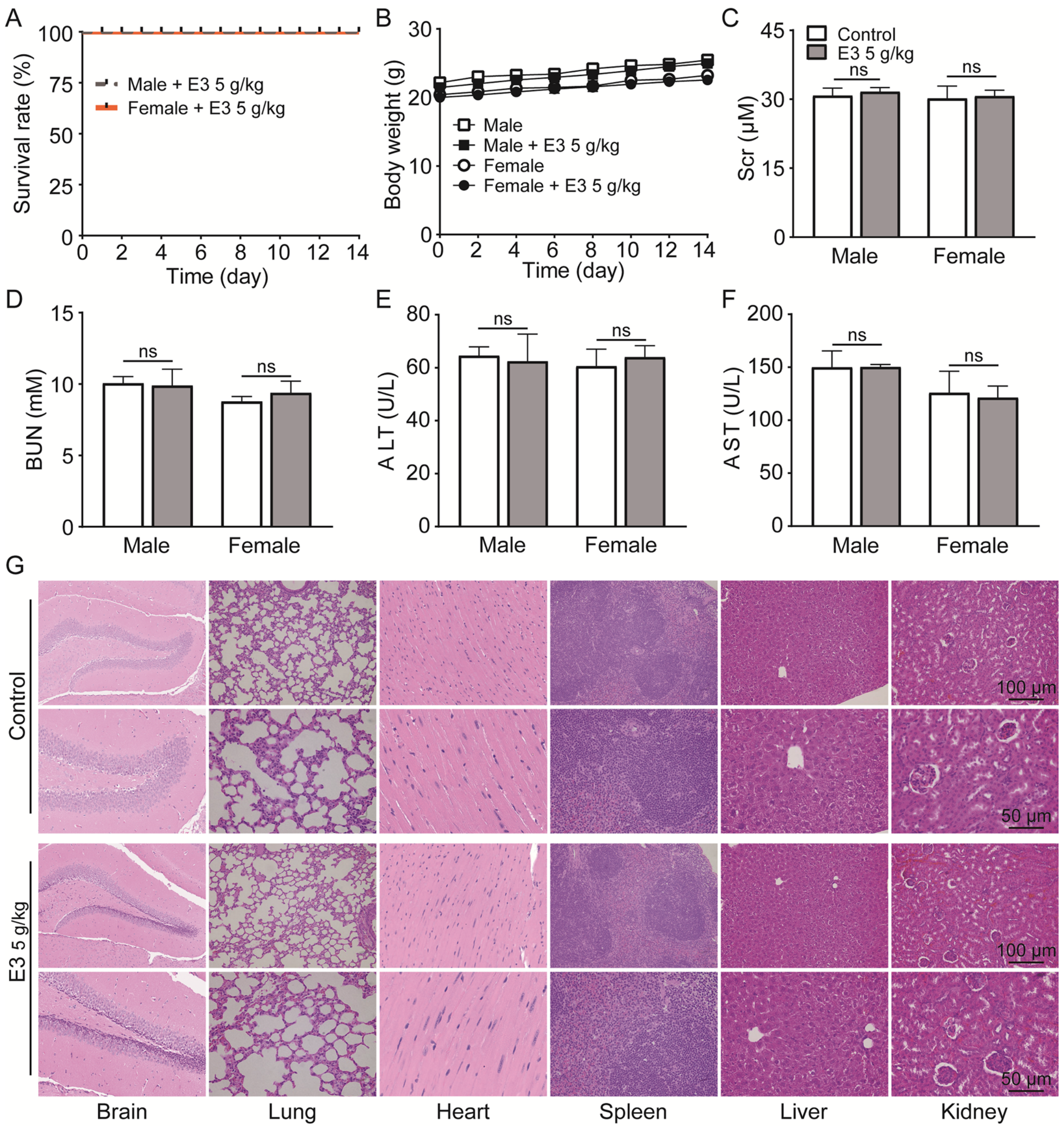

Figure S3. Acute oral toxicity assay in mice. (A) Survival rate. (B) Body weight. (C) Scr. (D) BUN. (E) ALT. (F) AST. (G) H&E staining of tissues from brain, lung, heart, spleen, liver, kidney. 100 μm scale bar (200× magnification) and 50 μm scale bar (400× magnification). Data are presented as mean ± SEM (n = 3). ns, no significance.

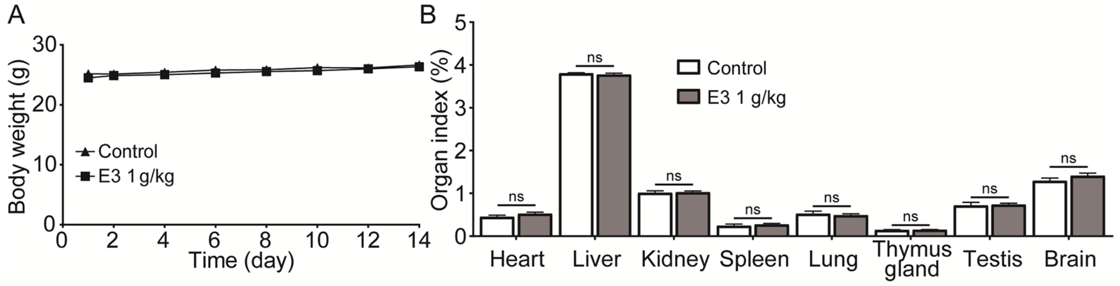

Figure S4. Subacute oral toxicity assay in recovery period. (A) Body weight changes of mice. (B) Organ indexes of mice. Data are presented as mean ± SEM (n = 3). ns, no significance.

Table S1. Body weight, organ indexes and blood chemistry in rats (n=6).

Measured parameters	Control	E3	
		4 mg/kg	20 mg/kg
Body weight (g)	247.6±7.1	242.4±2.3	240.8±4.1
Kidney index (%)	0.76±0.03	0.78±0.03	0.73±0.03
Heart index (%)	0.38±0.01	0.38±0.01	0.40±0.01
Liver index (%)	3.89±0.13	3.98±0.08	3.99±0.06
Spleen index (%)	0.27±0.01	0.30±0.02	0.29±0.01
Brain index (%)	0.57±0.02	0.58±0.01	0.64±0.01
Testis index (%)	0.85±0.03	0.88±0.06	0.94±0.02
Serum urea (mM)	7.31±0.45	7.15±0.16	7.18±0.19
Serum creatinine (μM)	34.3±1.4	33.6±1.7	34.1±1.6
ALT (U/L)	41.5±3.0	42.2±5.5	40.2±1.9
AST (U/L)	116.4±14.5	113.0±10.6	112.9±4.5
Serum Na (mM)	141.7±1.2	140.7±1.2	141.3±1.0
Serum K (mM)	5.21±0.16	5.16±0.10	4.98±0.12
Serum Cl (mM)	98.3±0.6	99.0±1.2	99.3±0.6

Table S2. Broader Profiles of E3 *in vitro* (n=3).

Property		Compound E3
ADME *in vitro* profiles		
Mouse/Rat/Rabbit/Human blood stability (remaining %)		45.3/46.8/35.7/54.1
Mouse colon/small intestine microbiota (remaining %)		89.3/87.3
Rat colon/small intestine microbiota (remaining %)		82.3/87.4
Mouse/Rat liver homogenate	Remaining (%)	39.8/69.0
	CL_{hep} (mL/min/kg)	99.3 /46.4
Safety		
hERG IC_{50} (μM)		> 33.3

Table S3. Organ indexes in mice in acute toxicity experiments (n=3).

Measured organ index (%)	Male		Female	
	**Control**	**E3 5g/kg**	**Control**	**E3 5g/kg**
Heart	0.50±0.003	0.5±0.01	0.48±0.03	0.52±0.04
Liver	5.05±0.03	5.16±0.12	4.92±0.17	4.86±0.08
Kidney	1.18±0.03	1.26±0.02	1.08±0.05	1.06±0.02
Spleen	0.35±0.004	0.37±0.02	0.35±0.01	0.4±0.01
Thymus	0.24±0.01	0.25±0.01	0.24±0.01	0.27±0.06
Brain	1.41±0.01	1.35±0.01	1.67±0.05	1.72±0.10
Lung	0.73±0.01	0.72±0.02	0.69±0.02	0.73±0.04
Testis/Uterus	0.73±0.01	0.73±0.02	0.32±0.01	0.3±0.02
Epididymis/Ovary	0.19±0.004	0.17±0.01	0.10±0.01	0.11±0.02

Table S4. Routine blood examination of subacute toxicity assay in mice (n=9).

Measured parameters	Control		E3 1 g/kg	
	Mean	SEM	Mean	SEM
WBC (10^9/L)	8.62	0.52	8.37	0.53
RBC (10^{12}/L)	8.17	0.11	8.03	0.06
HB (g/L)	81.67	1.12	80.11	0.66
HCT (%)	42.03	0.68	41.04	0.38
MCV (fL)	51.42	0.32	51.09	0.28
MCH (Pg)	9.99	0.14	9.96	0.12
MCHC (g/L)	194.67	3.08	195.33	2.05
RDW (%)	15.94	0.29	16.07	0.15
PLT (10^9/L)	1387.89	44.33	1313.22	13.70
MPV (fL)	2.88	0.03	3.01	0.02
NEU (%)	21.22	1.99	22.71	1.50
LYM (%)	75.54	1.57	74.12	1.59
EOS (%)	0.11	0.02	0.13	0.02
MONO (%)	3.11	0.52	3.02	0.25

WBC, white blood cell; RBC, red blood cell; HB, haemoglobin; HCT, hematocrit; MCV, mean corpuscular volume; MCH, mean corpuscular hemoglobin; MCHC, mean corpuscular hemoglobin concentration; RDW, red blood cell distribution width; PLT, platelet; MPV, mean platelet volume; NEU, neutrophil; LYM, lymphocyte; Eos, eosinophilic granulocytes; MONO, monocytes.

NMR spectrum of the target compounds.

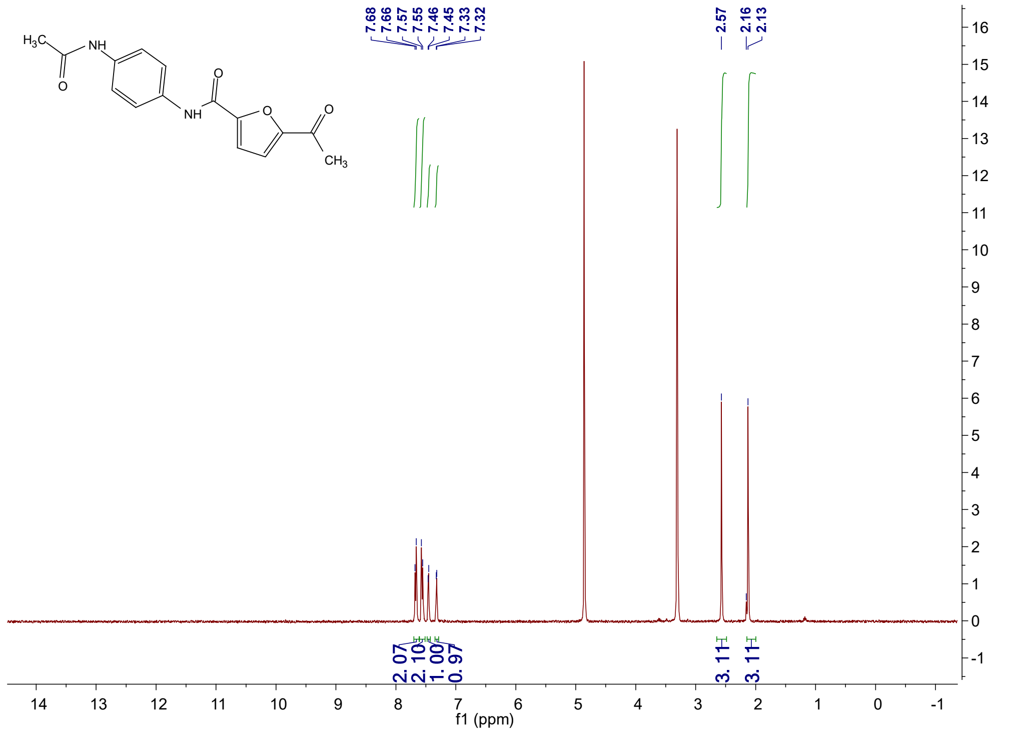

^{1}H NMR of compound **25a** (MeOD, 400 MHz)

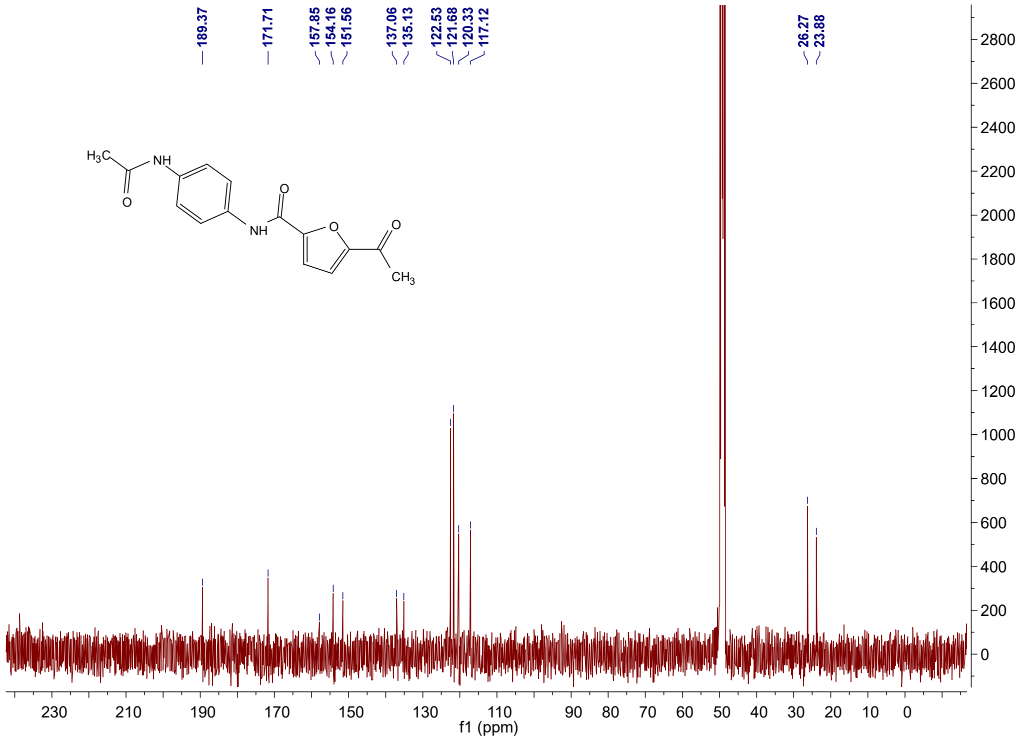

^{13}C NMR of compound **25a** (MeOD, 101 MHz)

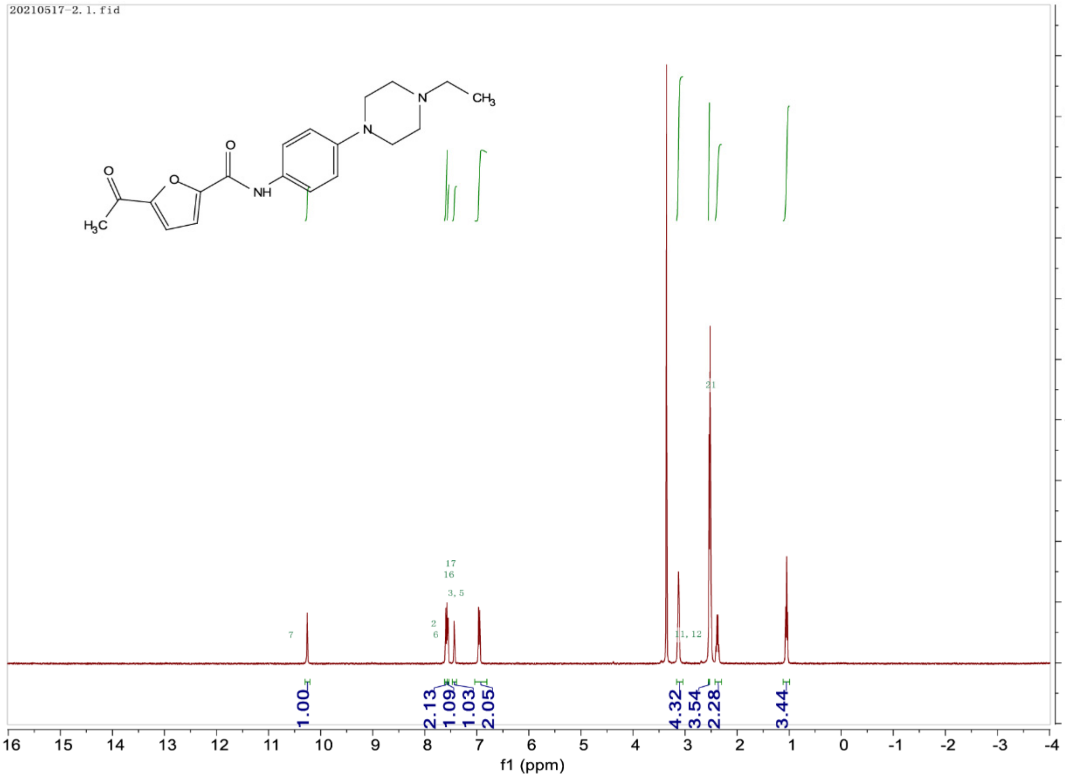

^{1}H NMR of compound **E1** (DMSO-d_6, 400 MHz)

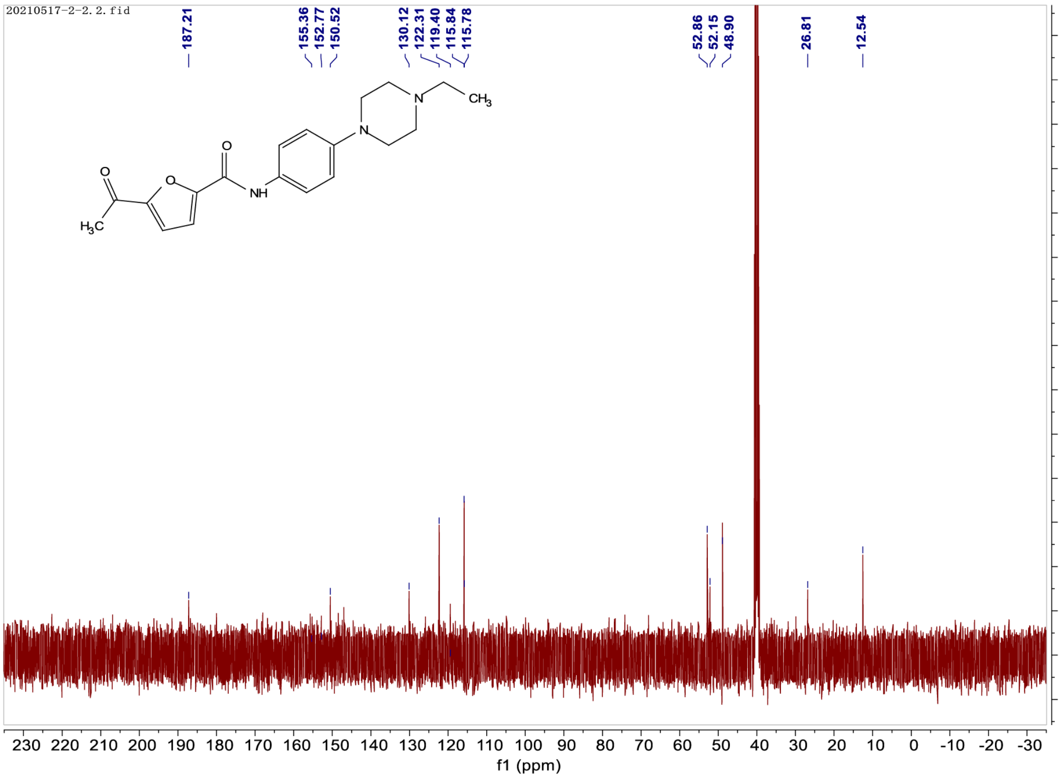

^{13}C NMR of compound **E1** (DMSO-d_6, 101 MHz)

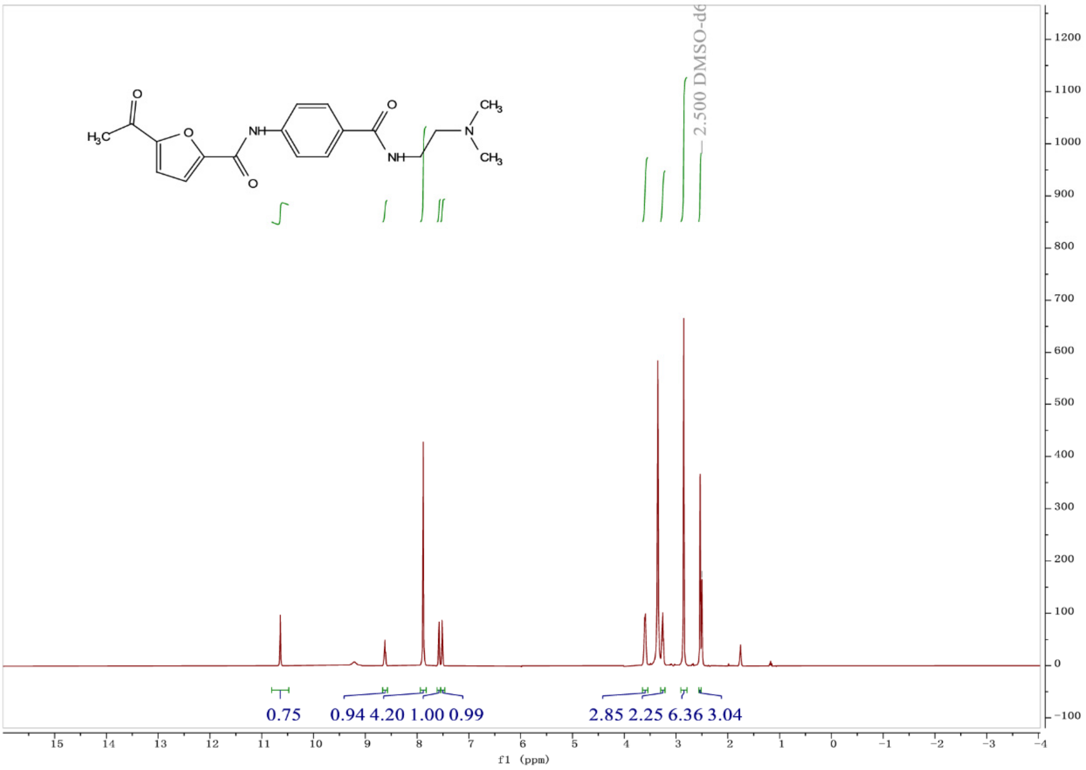

^{1}H NMR of compound **E2** (DMSO-*d₆*, 400 MHz)

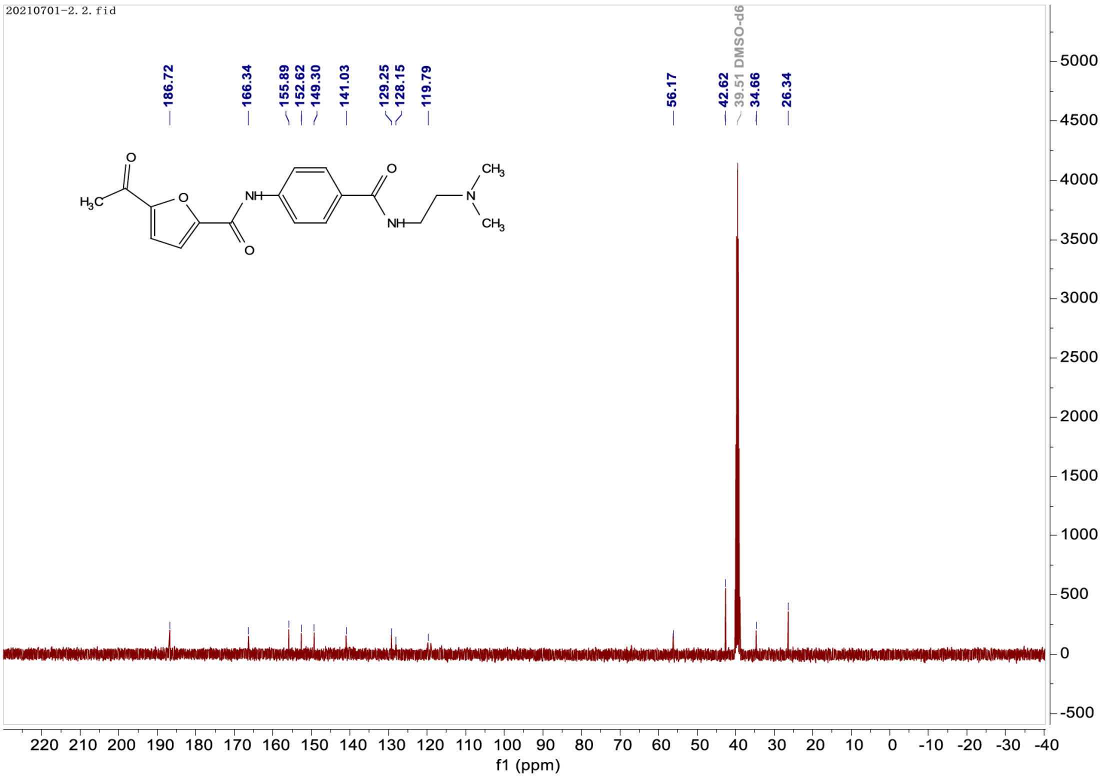

^{13}C NMR of compound **E2** (DMSO-*d₆*, 101 MHz)

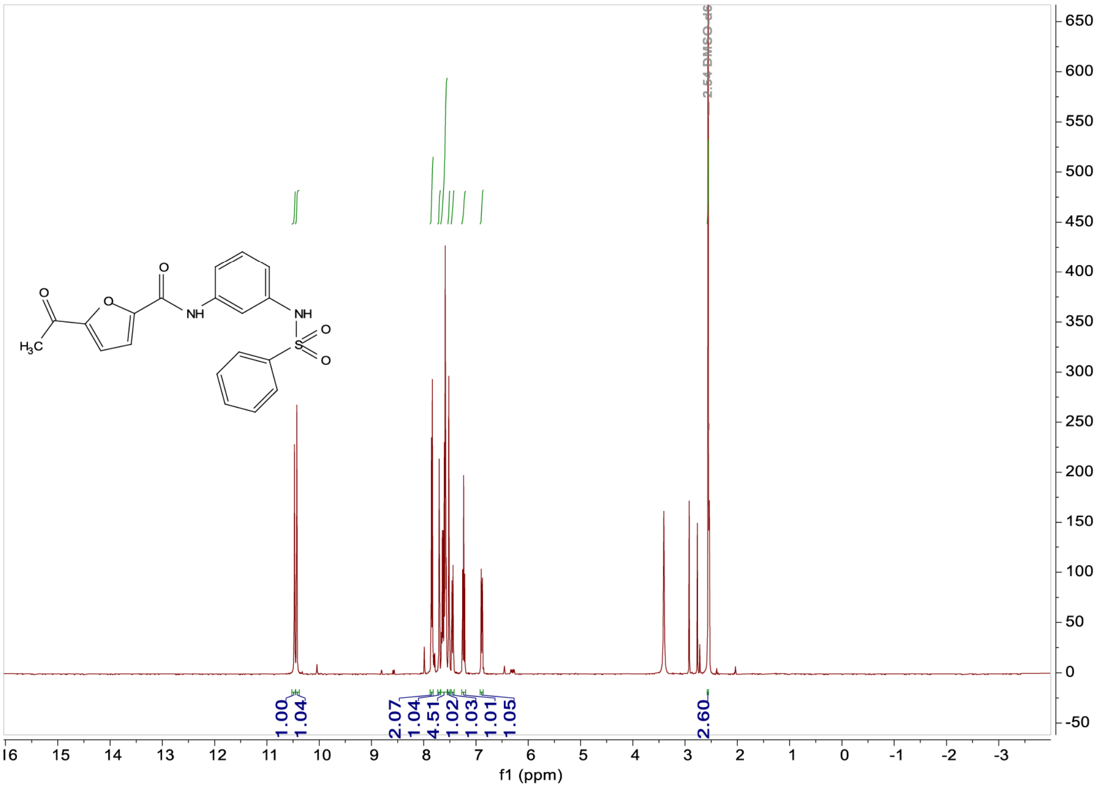

^{1}H NMR of compound **E3** (DMSO-d_6, 400 MHz)

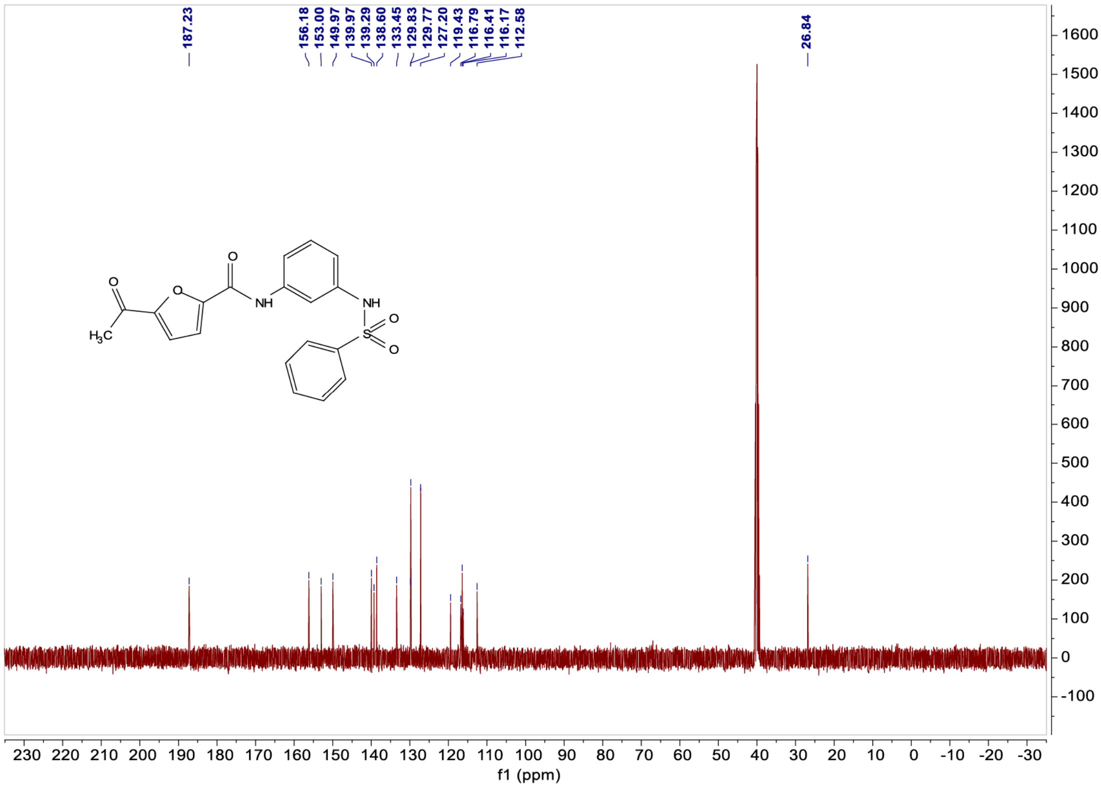

^{13}C NMR of compound **E3** (DMSO-d_6, 101 MHz)

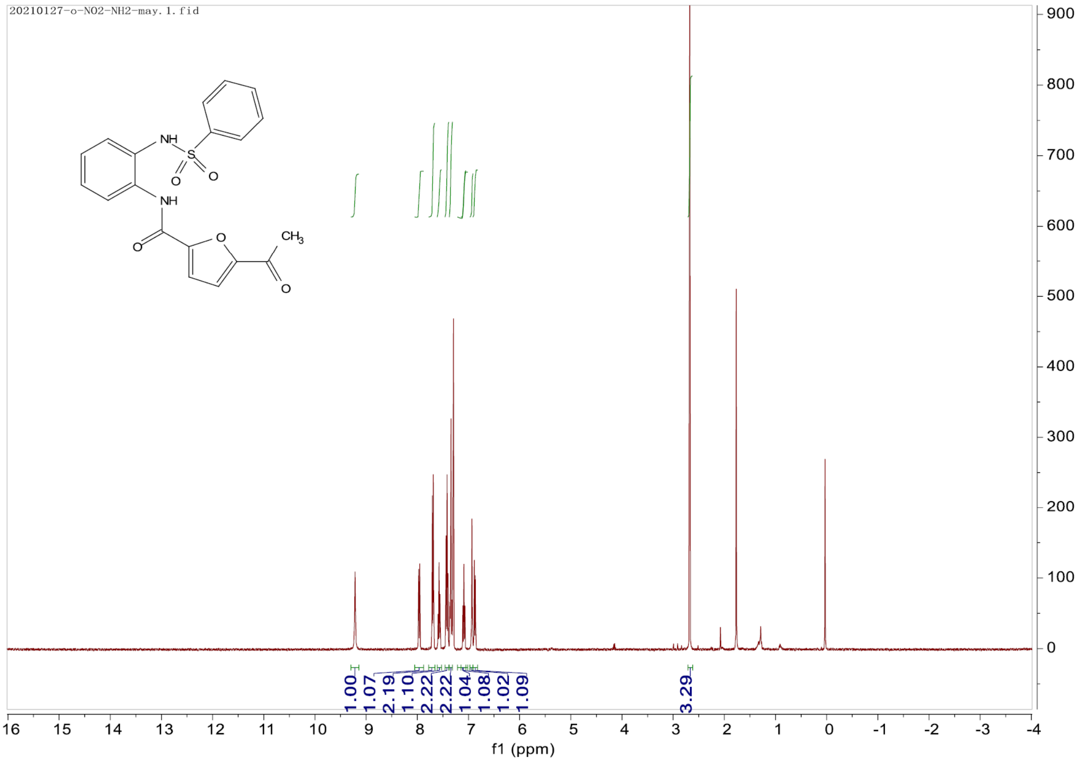

^{1}H NMR of compound **E4** ($CDCl_3$, 400 MHz)

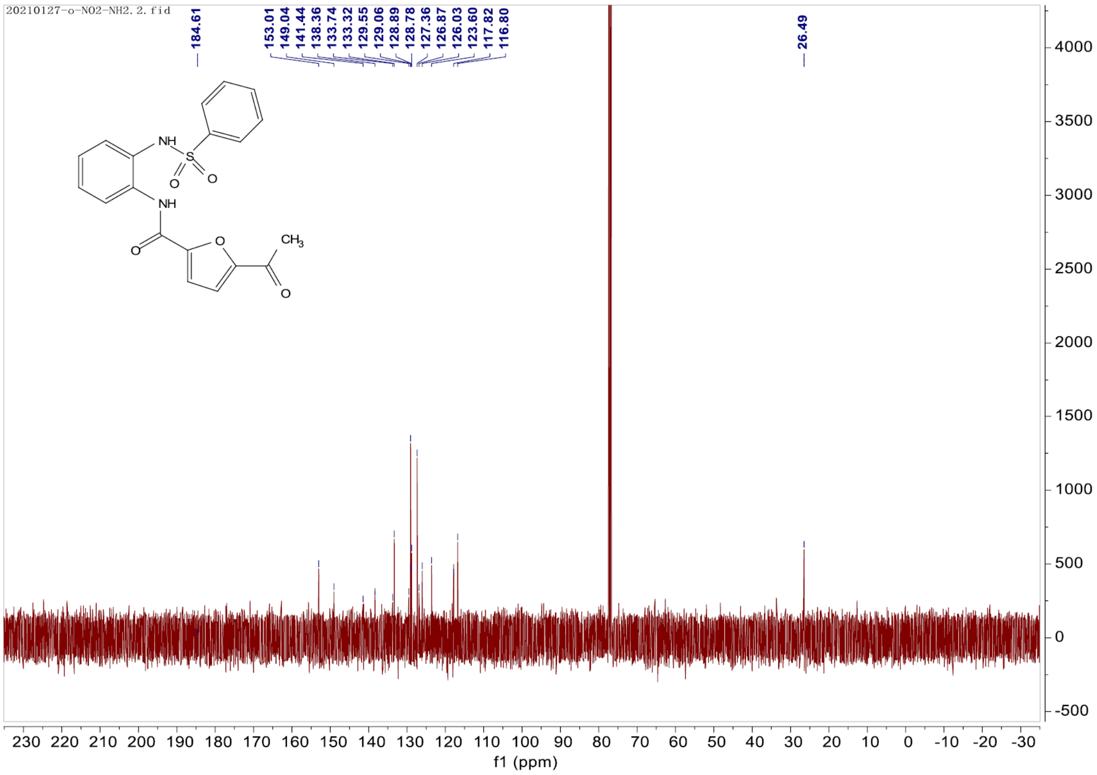

^{13}C NMR of compound **E4** ($CDCl_3$, 101 MHz)

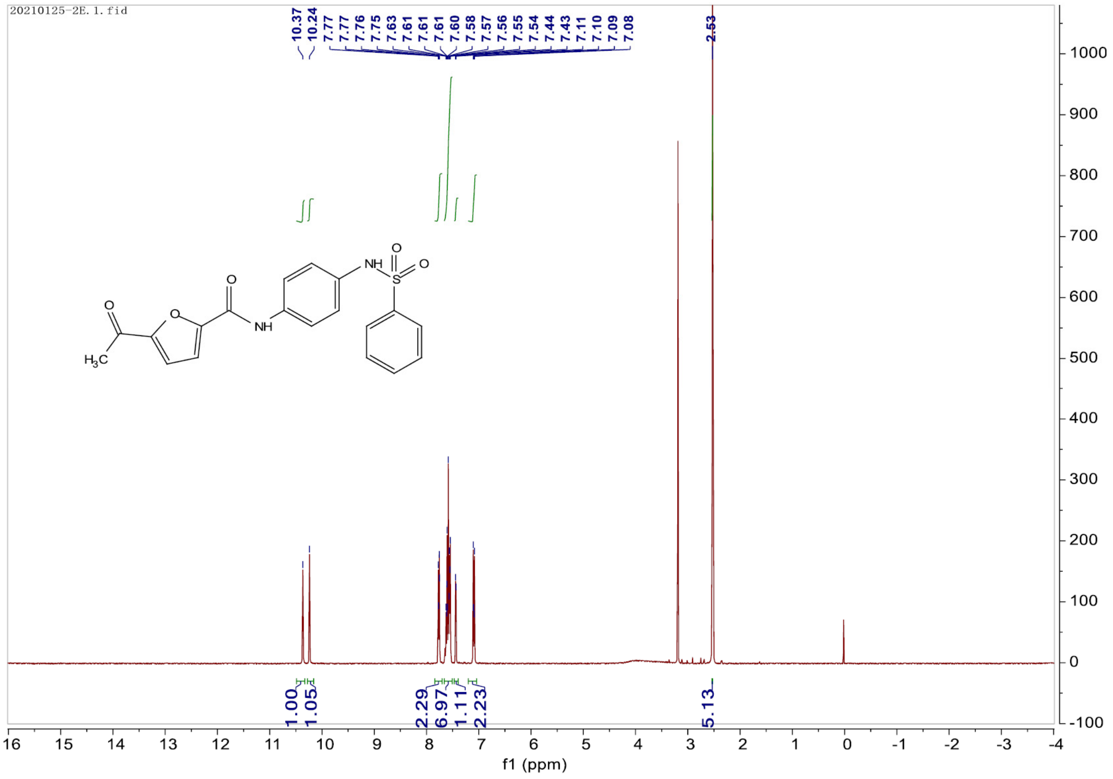

^{1}H NMR of compound **E5** (DMSO-*d₆*, 400 MHz)

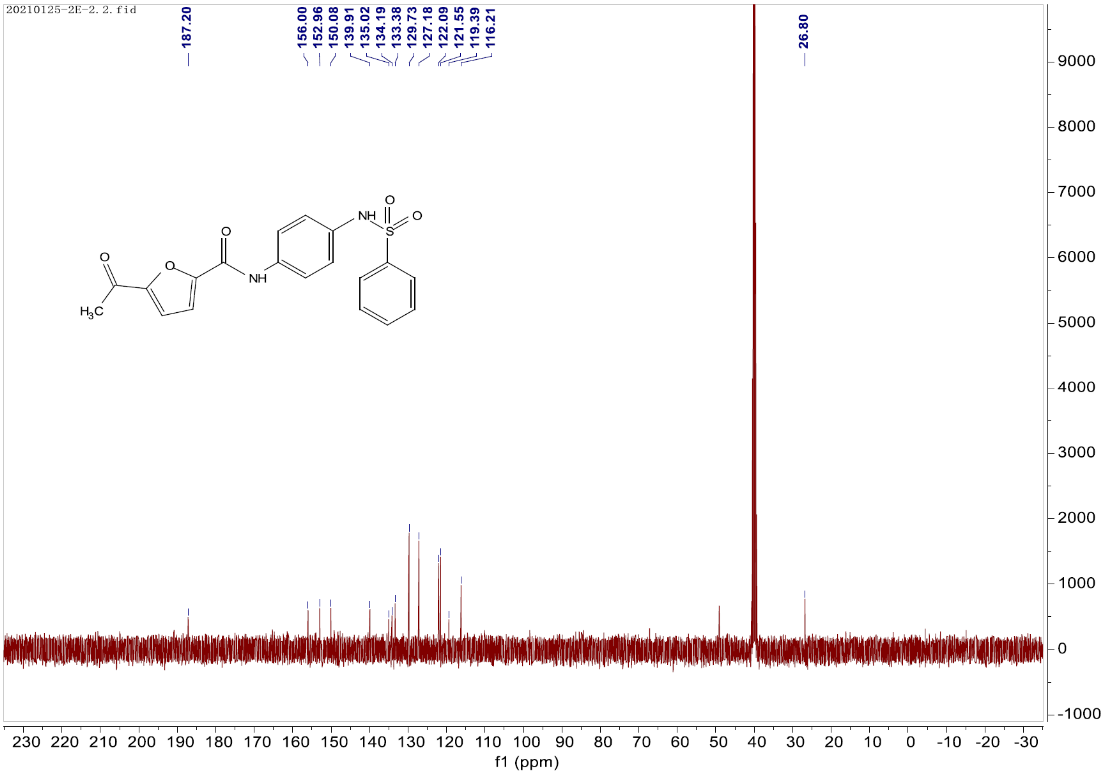

^{13}C NMR of compound **E5** (DMSO-*d₆*, 101 MHz)

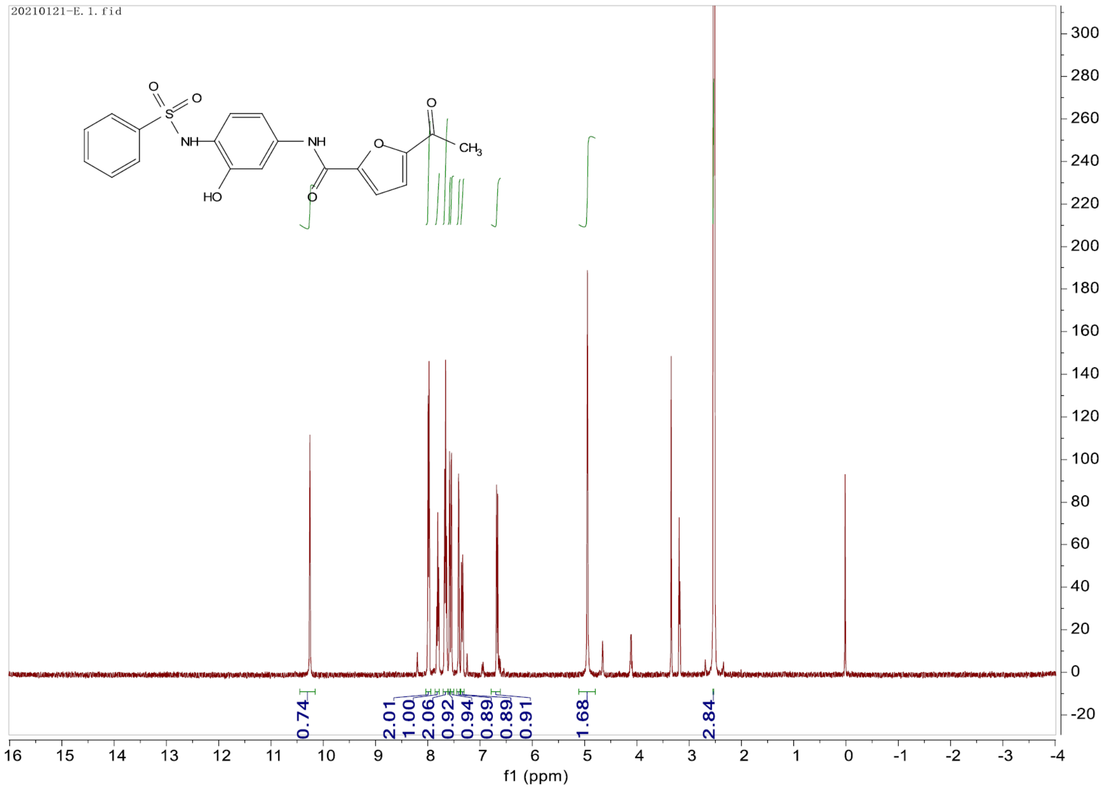

^{1}H NMR of compound **E6** (DMSO-*d₆*, 400 MHz)

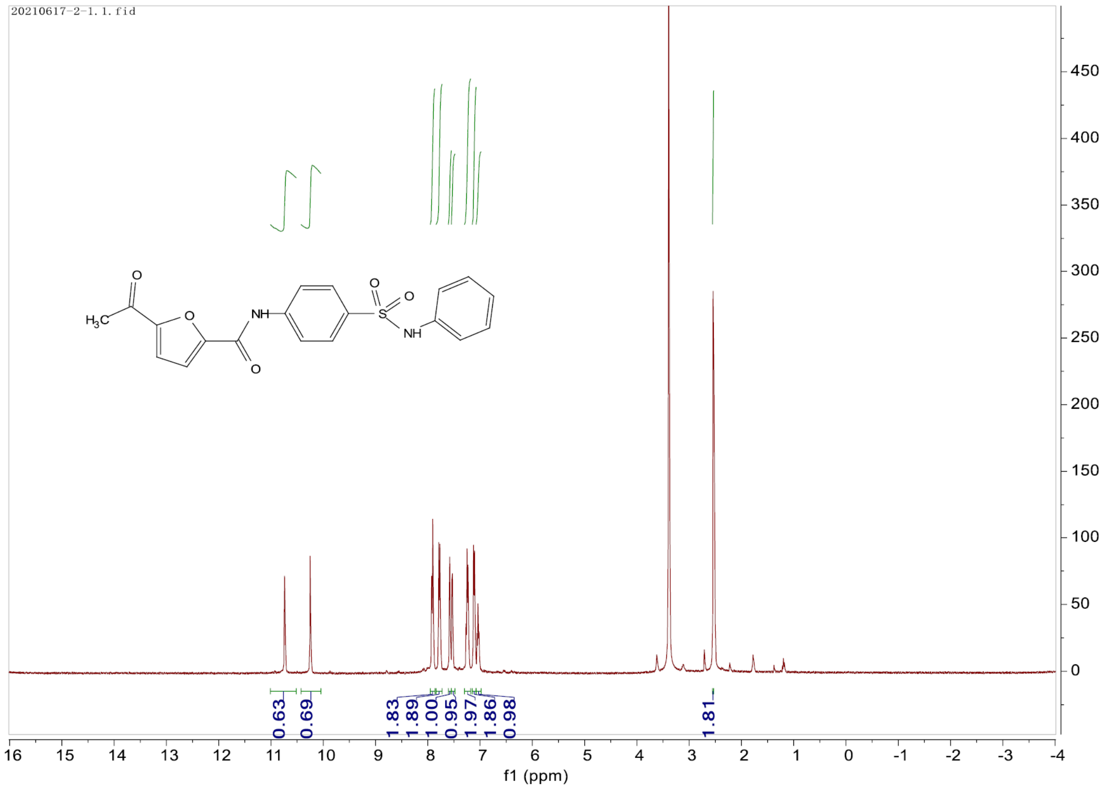

^{1}H NMR of compound **E7** (DMSO-*d₆*, 400 MHz)

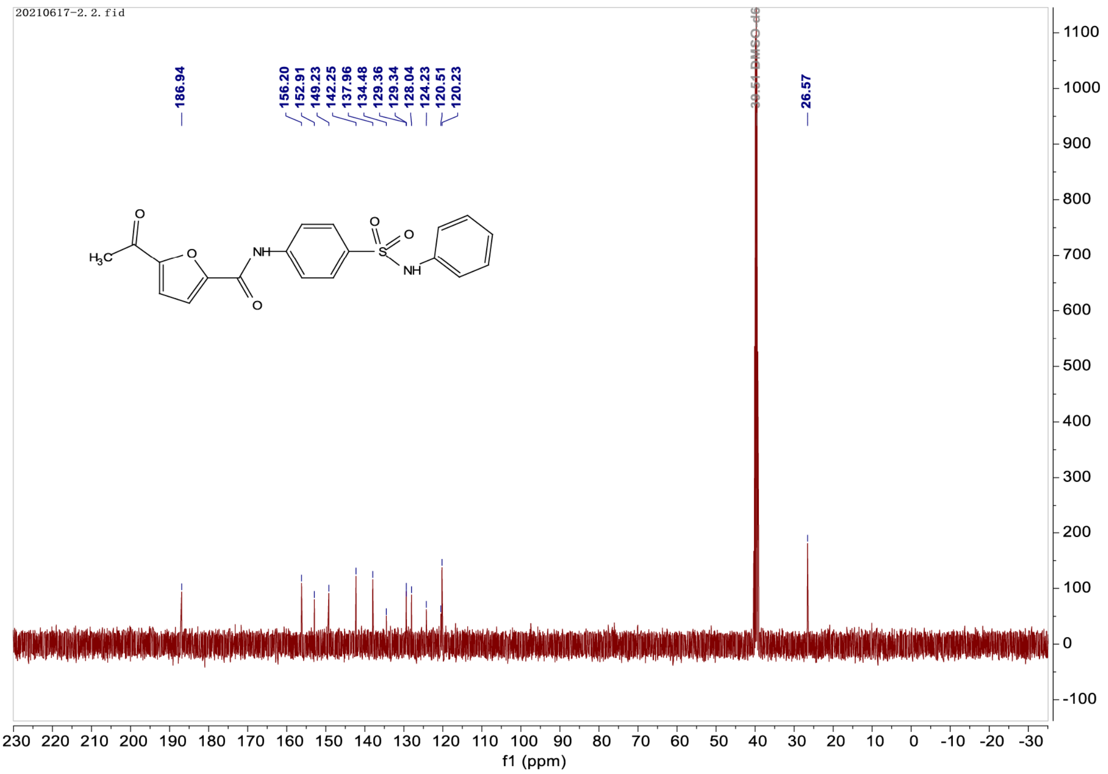

^{13}C NMR of compound **E7** (DMSO-*d*$_6$, 101 MHz)

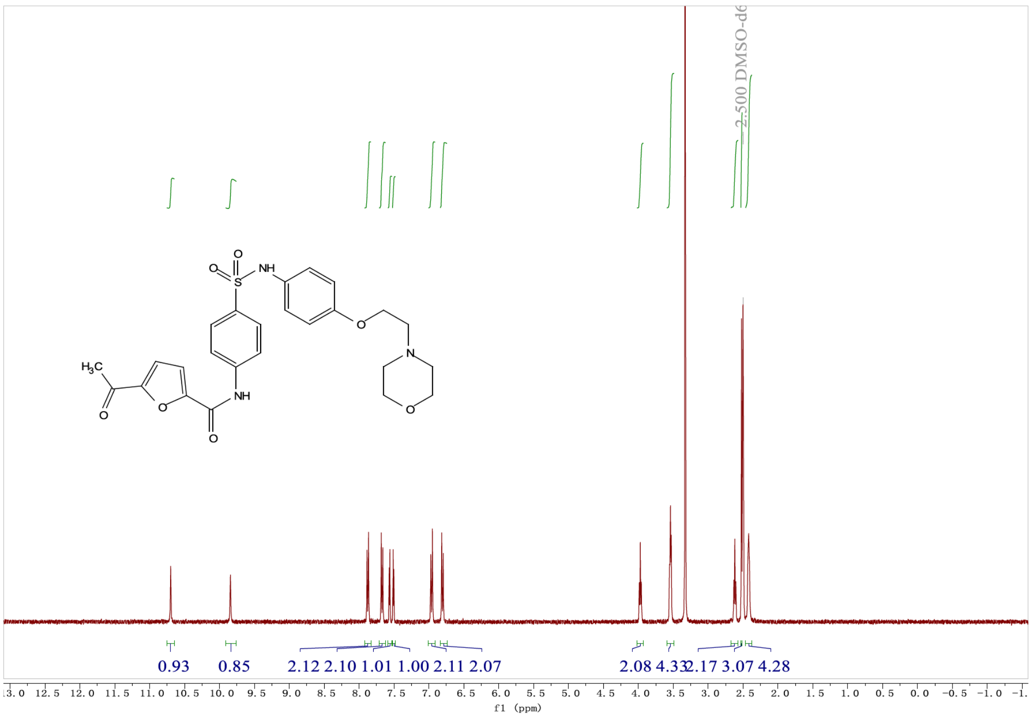

^{1}H NMR of compound **E8** (DMSO-*d*$_6$, 400 MHz)

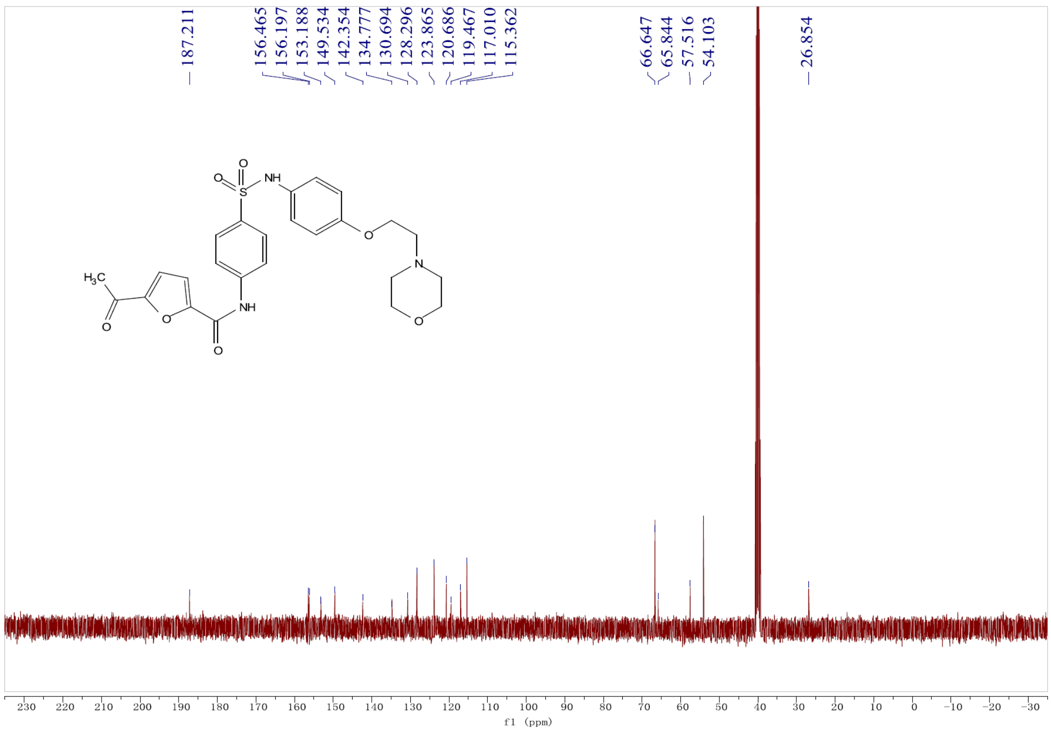

^{13}C NMR of compound **E8** (DMSO-d_6, 101 MHz)

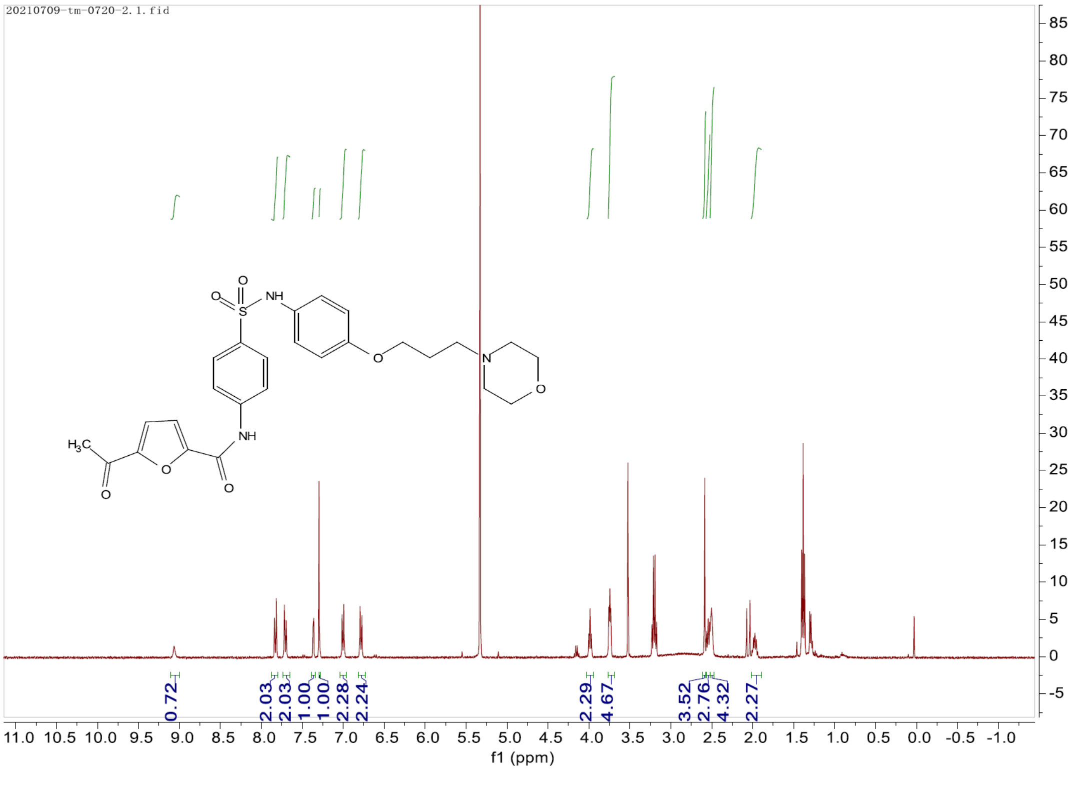

^{1}H NMR of compound **E9** ($CDCl_3$, 400 MHz)

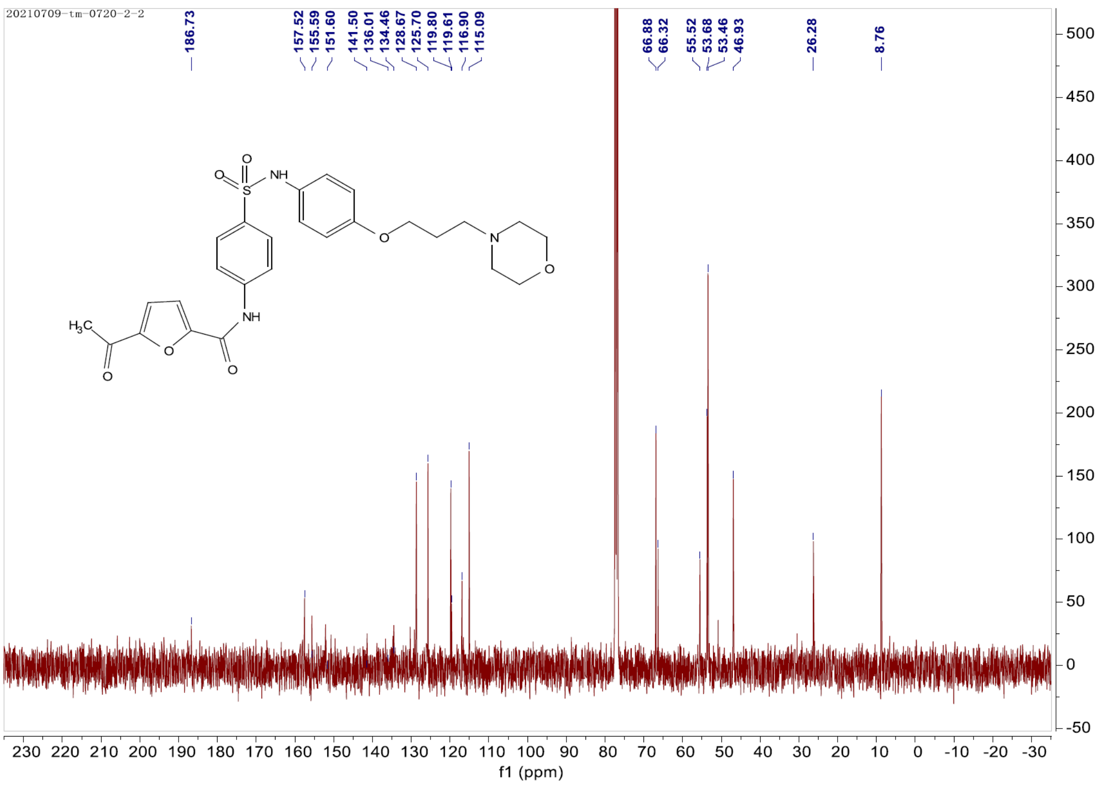

^{13}C NMR of compound **E9** ($CDCl_3$, 101 MHz)

HPLC chromatogram of compound **E3**.

Sample Name: E3-220607

```
=====================================================================
Acq. Operator   : SYSTEM
Sample Operator : SYSTEM
Acq. Instrument : LC1260                        Location : Vial 42
Injection Date  : 6/7/2022 1:51:40 PM
                                             Inj Volume : 1.000 µl
Acq. Method     : C:\CHEM32\1\METHODS\WSY20220406E3.M
Last changed    : 6/7/2022 1:25:21 PM by SYSTEM
                  (modified after loading)
Analysis Method : C:\CHEM32\1\METHODS\LY-HCL-NH-TPLP.M
Last changed    : 6/6/2022 3:33:46 PM by SYSTEM
                  (modified after loading)
```

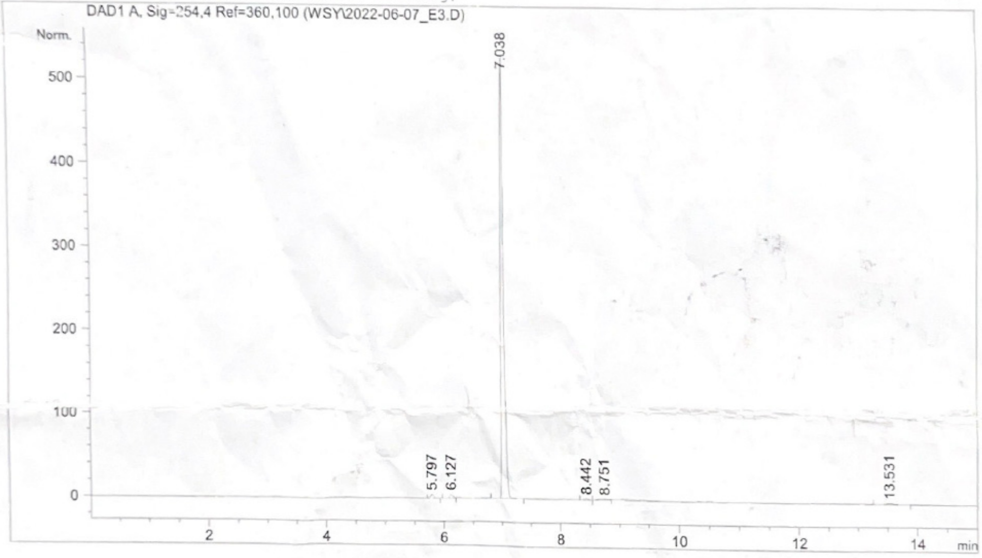


```
=====================================================================
                     Area Percent Report
=====================================================================

Sorted By             :      Signal
Multiplier            :      1.0000
Dilution              :      1.0000
Sample Amount:               :      1.00000  [ng/ul]   (not used in calc.)
Do not use Multiplier & Dilution Factor with ISTDs

Signal 1: DAD1 A, Sig=254,4 Ref=360,100
```

Peak #	RetTime [min]	Type	Width [min]	Area [mAU*s]	Height [mAU]	Area %
1	5.797	VB	0.0506	16.02717	4.84599	0.8790
2	6.127	BV	0.0536	16.64491	4.90496	0.9129
3	7.038	BB	0.0526	1760.20032	532.04718	96.5414
4	8.442	BB	0.0623	6.29179	1.58624	0.3451
5	8.751	BV	0.1681	13.92835	1.06870	0.7639
6	13.531	BB	0.1301	10.16797	1.16232	0.5577
Totals :				1823.26051	545.61539	
